# Using Proteomic Approaches to Unravel the Response of *Ctenocephalides felis felis* to Blood Feeding and Infection With *Bartonella henselae*

**DOI:** 10.3389/fcimb.2022.828082

**Published:** 2022-01-28

**Authors:** Marcos Rogério André, Pradeep Neupane, Michael Lappin, Brian Herrin, Vicki Smith, Taufika Islam Williams, Leonard Collins, Hongxia Bai, Gabriel Lemes Jorge, Tiago Santana Balbuena, Julie Bradley, Ricardo G. Maggi, Edward B. Breitschwerdt

**Affiliations:** ^1^ Laboratory of Immunoparasitology, Department of Pathology, Reproduction and One Health, Faculdade de Ciências Agrárias e Veterinárias, Universidade Estadual Paulista (FCAV/UNESP), Jaboticabal, Brazil; ^2^ Intracellular Pathogens Research Laboratory, Department of Clinical Sciences, The Comparative Medicine Institute, College of Veterinary Medicine, North Carolina State University, Raleigh, NC, United States; ^3^ Department of Clinical Sciences, Center for Companion Animal Studies, Colorado State University, Fort Collins, CO, United States; ^4^ Department of Diagnostic Medicine/Pathobiology, College of Veterinary Medicine, Kansas State University, Manhattan, KS, United States; ^5^ Department of Chemistry, North Carolina State University, Raleigh, NC, United States; ^6^ Molecular Education, Technology and Research Innovation Center (METRIC), North Carolina State University, Raleigh, NC, United States; ^7^ Departmento de Biotecnologia Agropecuária e Ambiental, Faculdade de Ciências Agrárias e Veterinárias, Universidade Estadual Paulista (FCAV/UNESP), Jaboticabal, Brazil

**Keywords:** bartonellosis, cat flea, cat scratch disease, flea-pathogen interface, proteome

## Abstract

Among the *Ctenocephalides felis felis*-borne pathogens, *Bartonella henselae*, the main aetiological agent of cat scratch disease (CSD), is of increasing comparative biomedical importance. Despite the importance of *B. henselae* as an emergent pathogen, prevention of the diseases caused by this agent in cats, dogs and humans mostly relies on the use of ectoparasiticides. A vaccine targeting both flea fitness and pathogen competence is an attractive choice requiring the identification of flea proteins/metabolites with a dual effect. Even though recent developments in vector and pathogen -omics have advanced the understanding of the genetic factors and molecular pathways involved at the tick-pathogen interface, leading to discovery of candidate protective antigens, only a few studies have focused on the interaction between fleas and flea-borne pathogens. Taking into account the period of time needed for *B. henselae* replication in flea digestive tract, the present study investigated flea-differentially abundant proteins (FDAP) in unfed fleas, fleas fed on uninfected cats, and fleas fed on *B. henselae*-infected cats at 24 hours and 9 days after the beginning of blood feeding. Proteomics approaches were designed and implemented to interrogate differentially expressed proteins, so as to gain a better understanding of proteomic changes associated with the initial *B. henselae* transmission period (24 hour timepoint) and a subsequent time point 9 days after blood ingestion and flea infection. As a result, serine proteases, ribosomal proteins, proteasome subunit α-type, juvenile hormone epoxide hydrolase 1, vitellogenin C, allantoinase, phosphoenolpyruvate carboxykinase, succinic semialdehyde dehydrogenase, glycinamide ribotide transformylase, secreted salivary acid phosphatase had high abundance in response of *C. felis* blood feeding and/or infection by *B. henselae*. In contrast, high abundance of serpin-1, arginine kinase, ribosomal proteins, peritrophin-like protein, and FS-H/FSI antigen family member 3 was strongly associated with unfed cat fleas. Findings from this study provide insights into proteomic response of cat fleas to *B. henselae* infected and uninfected blood meal, as well as *C. felis* response to invading *B. henselae* over an infection time course, thus helping understand the complex interactions between cat fleas and *B. henselae* at protein levels.

## Introduction

Cat fleas (*Ctenocephalides felis felis*) (Bouché, 1835) belong to the Order Siphonaptera and the family Pulicidae. Although found throughout much of the world, the geographical distribution of *C. felis* continues to expand, as does the number of hosts infested with this insect. Recently, with increased temperatures associated with global warming, it has been proposed that the number of generations per year and potential density of cat fleas might dramatically increase (Rust, 2017). Even though *C. felis* has been found parasitizing dogs and several wild animals, several reports confirm that cats are more often infested by this flea species than dogs (Rust, 2017). Despite the prevalence of *C. felis* being seasonal, this flea species appears throughout the year. In the US, about 65% of all households have companion animals, mostly cats and dogs. Fleas and ticks comprise a considerable pet welfare concern and the annual investment by owners in preventive acaracide and insecticde products is substantial. It was estimated that 2.4 billion US dollars was spent on ectoparasiticides for companion animals in 2011 (Rust, 2017).

Cat fleas may cause direct damage to the skin, discomfort, nuisance, allergic reactions, anemia, and can transmit zoonotic pathogens, such as *Rickettsia typhi*, *Rickettsia felis* and *Bartonella* species, including *Bartonella henselae, Bartonella clarridgeae* and *Bartonella koehlerae* (Traversa, 2013; Bouhsira et al., 2013; Nogueras et al., 2013; Angelakis et al., 2016). *Ctenocephalides felis* is also a competent intermediate host for the tapeworm *Dipylidium caninum* and the filarial nematode *Acanthocheilonema reconditum* (Fourie et al., 2012; Traversa, 2013).

Among the *C. felis*-borne pathogens, *Bartonella henselae*, the main aetiological agent of cat scratch disease (CSD), is of increasing comparative biomedical importance. Besides CSD, *B. henselae* has also been associated with endocarditis, osteomyelitis, neuroretinitis and neuropsychiatric symptoms in immunocompetent persons, and bacillary angiomatosis, bacillary peliosis and endocarditis in immunocompromised individuals (Breitschwerdt and Kordick, 2000; Chomel, 2000; Breitschwerdt et al., 2020; Lashnits et al., 2021). Transmission of *B. henselae* from cats to human beings may occur through contamination of cat scratches with flea excrement (Foil et al., 1999). Transmission may also occur through cat bites if cat blood or flea excrement contaminates the bite site and viable organisms are in eyrthrocytes in the oral cavity of the cat (Guptill, 2010).

*Bartonella henselae* is naturally transmitted by *C. felis* among cats (Chomel et al., 1996), which represent the bacteria’s main vertebrate reservoir (Breitschwerdt and Kordick, 2000). Bacteremia with *B. henselae* is usually chronic, accompanied by a relapsing bactermia in cats. Experimentally infected cats in arthropod-free environments maintained relapsing *B. henselae* bacteremia for as long as 454 days, with relapses of bacteremia occurring at irregular intervals between 1 and 4.5 months (Kordick et al., 1999), which likely favors persistence of the bacteria in cats, as well as the transmission by arthropod-vectors (Breitschwerdt et al., 2010).

Previously, *B. henselae* was transmitted among cats in laboratory studies by transferring fleas fed on naturally infected cats to specific pathogen-free cats, and by intradermal inoculation of excrement from infected fleas (Chomel et al., 1996; Foil et al., 1999). The administration of 10% imidacloprid/1% moxidectin and 10% imidacloprid/4.5% flumethrin was shown to block transmission of *B. henselae* amongst cats experimentally exposed to infected *C. felis* (Bradbury and Lappin, 2010; Lappin et al., 2013). Data has clearly shown the persistence of viable *Bartonella* organisms, as well as an increase in *Bartonella* bacterial load, in flea guts 9 days after ingesting an infected blood meal (Higgins et al., 1996). Replication of *B. henselae* within adult fleas at 6 to 8 days after ingestion of a blood meal was also documented (Finkelstein et al., 2002). There was a decrease in *B. henselae* DNA in fleas 2 days after ingesting an infected blood meal, which may have been due to fleas clearing themselves of an excess of bacteria in conjunction with digested blood waste products, after which bacterial numbers in flea guts increased (Higgins et al., 1996; Bouhsira et al., 2013). This bacterial pathogen is able to survive for at least 3 days in flea feces, suggesting that “flea dirt” is an important source of environmental contamination (Filkelstein et al., 2002). Since cats did not become infected with *B. henselae* when fed on by *Bartonella*-infected fleas enclosed in capsules attached to the skin that prevented inoculation of infected flea excrement by scratching or grooming, transmission does not appear to occur *via* flea saliva (Foil et al., 1999).

Despite the importance of *B. henselae* as an emergent pathogen, prevention of the diseases caused by this agent in cats, dogs and humans mostly relies on the use of ectoparasiticides that kill or inhibit the growth of cat fleas. However, flea control is difficult and requires integrated approaches combining insecticides, such as adulticides (e.g. Fipronil, Spinosad, Selamectin, Afoxolaner, Fluralaner, Sarolaner), insect growth inhibitors (e.g. Lufenuron), and juvenile hormone analogues (e.g. S-methoprene), and treatment of animals may be necessary to eliminate flea infestations (Marsella, 1999; Siak and Burrows, 2013; Halos et al., 2014; Becskei et al., 2017; Rust, 2017; Vatta et al., 2017). In addition, resistance or reduced efficacy of ectoparasiticde products has been reported for some historically efficacious compounds (Traversa, 2013; Coles and Dryden, 2014; Rust, 2017), emphasizing the need for the identification of efficient, alternative, and environmentally friendly approaches.

Vaccination would represent a promising and sustainable alternative measure for the control of fleas and flea-borne pathogens in cats and dogs. A vaccine targeting both flea fitness and pathogen competence is an attractive choice requiring the identification of flea proteins/metabolites with a dual effect. Even though recent developments in vector and pathogen omics have advanced the understanding of the genetic factors and molecular pathways involved at the tick-pathogen interface, leading to discovery of candidate protective antigens (de la Fuente et al., 2007a; de la Fuente et al., 2007b), only a few studies have focused on the interaction between fleas and flea-borne pathogens (Dreher-Lesnick et al., 2010; Brown, 2019; Bland et al., 2020; Danchenko et al., 2021; Laukaitis and Macaluso, 2021).

Therefore, to better understand interactions among flea and pathogen biology, pathogen transmission dynamics, and new potential control or preventive approaches, it was desirable to gain more knowledge about the relationship between *C. felis* and *B. henselae*. In *C. felis*, the digestive tract plays a key interactive role between the bacteria and the vector, thereby influencing pathogen transmission to animals and humans.

The underlying principles of this study were based on the following facts:

i.) Studies involving the molecular mechanisms of *Bartonella* infection in vectors are important for the development of safe and effective control strategies; ii.) Vaccine candidates targeting both the pathogen and the flea would provide a better approach for the control of flea infestations and the prevention of *B. henselae* transmission; iii.) Analysis of proteins differentially expressed among three different *C. felis* populations (uninfected and unfed, uninfected and fed, and *B. henselae*-infected) would potentially allow for the selection of proteins related with processes of *B. henselae* transmission and infection of various hosts.

Taking into account the period of time needed for *B. henselae* replication in flea digestive tract (Finkelstein et al., 2002), the present study investigated flea-differentially abundant proteins (FDAP) in unfed fleas, fleas fed on uninfected cats, and fleas fed on *B. henselae*-infected cats at 24 hours and 9 days after the beginning of blood feeding. Proteomics approaches were designed and implemented to interrogate differentially expressed proteins, so as to gain a better understanding of proteonomic changes associated with the initial *B. henselae* transmission period (24 hour timepoint) and a subsequent time point 9 days after blood ingestion and flea infection.

## Material and Methods

### Experiment Outline

**Figure 1:**
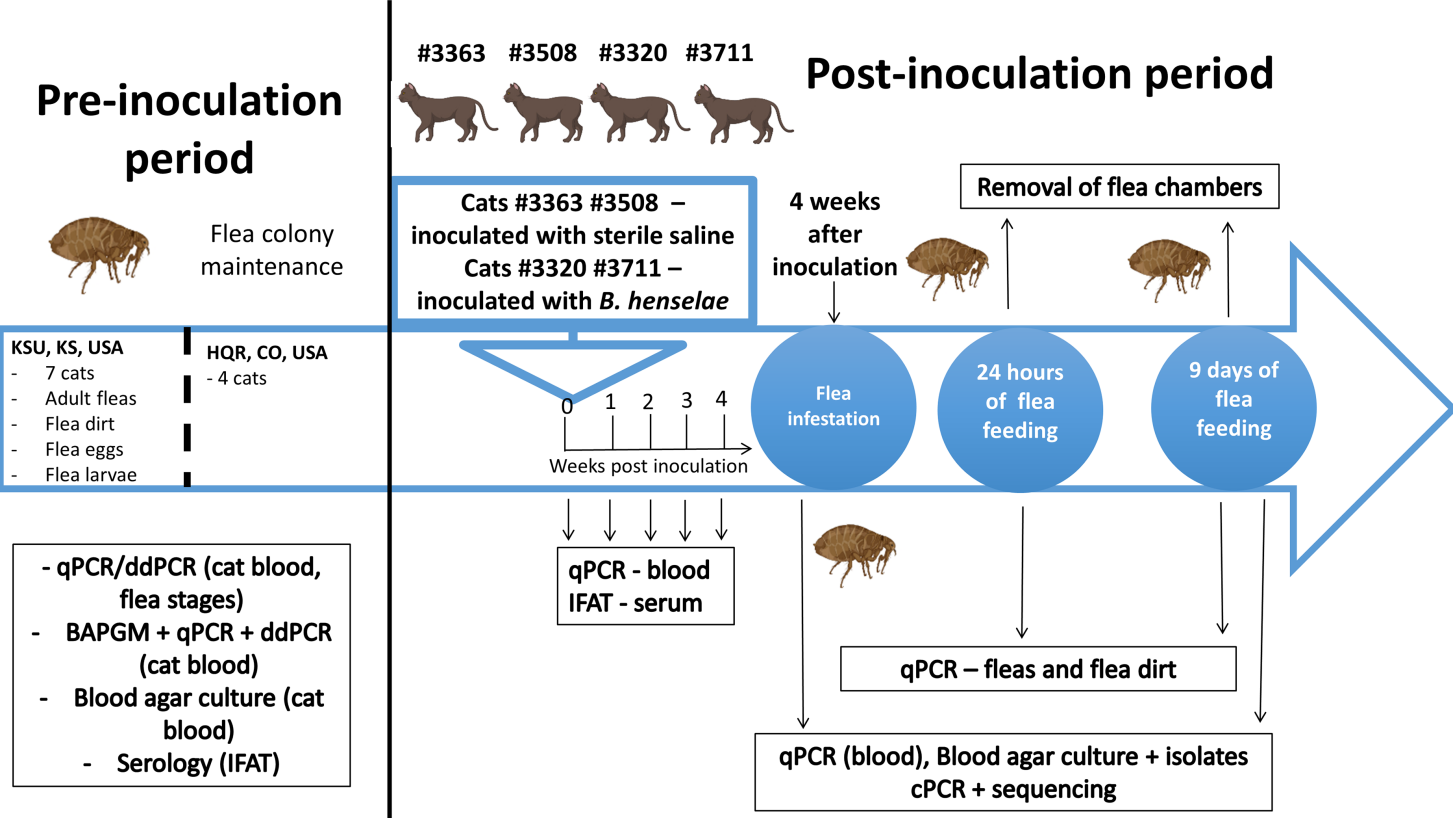
Schematic timeline of the flea proteomic experimental study. Created with Biorender.com.

*Ctenocephalides felis* used in this study were laboratory-reared at Kansas State University (KSU), Manhattan, KS. To generate the large number of fleas needed for this study, 7 adult cats maintained in the referred institution under KSU IACUC #4511-VMS were used for flea feeding, with the aim to produce eggs, larvae, pupae and new-emerged adults for maintenance and expanding of the flea colony. Prior to their use in this experiment, blood samples from the seven cats were submitted for testing using serology (IFAT –Immunofluorescent Antibody Test), real-time PCR (qPCR), droplet digital PCR (ddPCR), and enrichment blood culture followed by qPCR and ddPCR assays in order to confirm the absence of *B. henselae* infection. Also, adult flea pools (up to 10 adult fleas), flea eggs, and larvae collected from the KSU flea colony were submitted for qPCR and ddPCR assays to confirm the absence of *Bartonella* DNA in the fleas maintained within the colony. These serological, molecular and microbiological tests were performed at the Intracellular Pathogens Research Laboratory, College of Veterinary Medicine, North Carolina State University, Raleigh, North Carolina, USA (**Figure 1**).

Similarly, blood samples from four cats maintained at High Quality Research (HQR), Fort Collins, Colorado, USA, under an approved protocol (number #170.059) – two cats to be infected with *B. henselae* and two to be used as negative, uninfected controls) were also tested using the above mentioned assays in order to confirm the absence of *B. henselae* infection in the cats to be experimentally infected and to be used as controls, respectively. Cats were housed in facilities free of arthropods, including fleas and ticks, and cared for according to the Animal Welfare and Protection Rules. The microbiological testing procedures, cat designations and experimental design are depicted in **Figure 1**.

### *Ctenocephalides felis* acquisition

As previously mentioned, *C. felis* used in this study were laboratory-reared at Kansas State University (KSU), Manhattan, KS. To generate the fleas needed for the study, seven cats were infested twice a week with 50 adult *C. felis*. Eggs were collected on a daily basis from the cage collection pan and sifted from debris using a series of sieves. The eggs were poured into a petri dish and covered with media, which consisted of autoclaved masonry sand and larval food that consisted of dried bovine blood, brewer’s yeast and ground dog food. The petri dish was placed into the incubator and maintained at approximately 28°C and 70% relative humidity. Larvae were sifted from the media, and pupae were placed into jars where they emerged into adults. A vacuum system was used to remove fleas from jars, after which they were counted. Fleas were transported in vials from KSU to the animal facility in Colorado for infestations on the two experimental cat groups.

### Experimental Infection of Cats With *B. henselae* (CSU Bh-1 Strain) and Infestation With *C. felis*

The four young adult female cats used in the *C. felis* feeding experiments were housed in an ectoparasite flea facility. Prior to experimental challenge with *B. henselae*, the cats were normal by physical examination, and tested negative for *B. henselae* bacteremia based upon enrichment blood culture (Maggi et al., 2005; Duncan et al., 2007) and 16S-23S internal transcribed spacer (ITS) region- based qPCR and a more sensitive ddPCR assay (Maggi et al., 2020). The attitude and appetite of individual cats was monitored daily, before and after *B. henselae* inoculation and *C. felis* feeding. A complete physical examination, including rectal body temperature and cardiac auscultation were to be performed if any abnormalities developed during the study. Following *B. henselae* intradermal inoculation, blood specimens were drawn from the jugular vein of each cat for isolation of *B. henselae*, using the techniques described below.

*Bartonella henselae* (CSU Bh1 strain) was cultured on blood agar at 35 oC in 5% CO_2_ and harvested at day 5-7. Inocula were quantified using the McFarland Standard method. Colonies of CSU Bh-1 were harvested and suspended in 1 ml of sterile saline at 0.5 McFarland, which is equivalent to approximately 1.5 X 10^8^ organisms. While sedated using the approved facility protocol, the cats were inoculated intradermally in 4 different sites (0.25 mL/site), as previously described (ABBOT et al., 1997). The two negative control (not infected with *B. henselae*) cats were inoculated with 1.0 mL of sterile saline. Blood (EDTA anticoagulant) and serum were collected from the cats weekly and stored at -80°C until assayed.

**Figure 2:**
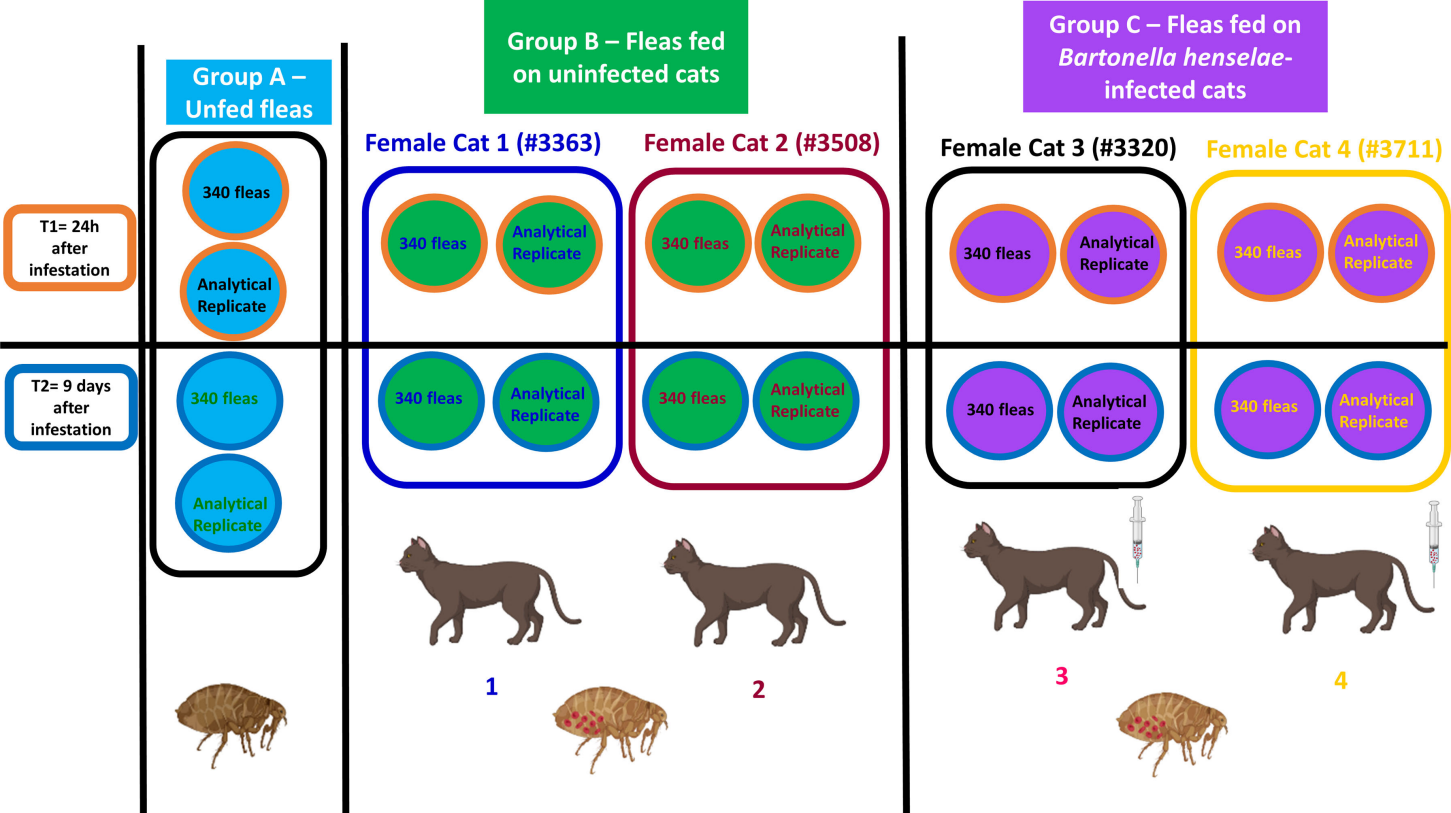
Experimental design used in this study for investigation of differentially abundant proteins in (Group A) unfed fleas, (Group B) fleas fed on uninfected cats and (Group C) fleas fed on *B. henselae*-infected cats. Fleas were collected from the cats at 24 hours and 9 days after infestation for proteomic analyses. Created with Biorender.com.

Once *B. henselae* bacteremia was confirmed, *C. felis* in chambers were attached to shaved skin of the sedated cats. The hair over the thorax and flank was shaved to allow the flea chamber feeding membrane to have direct contact the skin. Two chambers were placed on both sides of each cat. The experimental study design is illustrated in **Figure 2**.

### Immunofluorescence Antibody Test (IFAT) for *Bartonella* spp.

An IFAT assay used for the detection of IgG antibodies to *B. henselae* in the serum of the experimental cats, following protocols previously described (Neupane et al., 2018).

### Blood Culture

Prior to the experiment (pre-infection period), cat blood samples (from seven cats from KSU - used for flea colony maintainance – and from four cats from HQR – two to be experimentally infected with *B. henselae* and two to be used as *B. henselae* negative controls) were cultured using a liquid growth medium, as previously described by Duncan et al. (2007) and Maggi et al. (2005), with some modifications. After the cat blood specimens were thawed, an aliquot of 500 μL was inoculated into filter cap cell culture flasks with 2 mL of liquid *Bartonella* alphaproteobacterium growth media (BAPGM). A negative-control flask (uninoculated BAPGM culture flask) was prepared simultaneously with each batch of cat blood cultures. All flasks were incubated at 35°C in 5% CO2 in a water-saturated atmosphere and maintained with a constant shaking motion for 7 (C7), 14 (C14) and 21 (C21) days. After incubation for 7 days, a 500 μL aliquot of the C7 liquid culture was plated on blood agar plates (Thermo Scientific), which were incubated at 35°C in 5% CO2 in a water-saturated atmosphere and examined weekly for *Bartonella* growth for up to 45 days. Aliquots from C7, C14 and C21 liquid cultures were submitted to DNA extraction and qPCR for *Bartonella* spp.

During the experiment (post-infection period), 500 μL of previously frozen blood (collected immediately prior to flea infestation and 9 days after) from the two CSU-1 *Bh*-experimentally infected cats and from the two cats inoculated with sterile saline solution (controls) maintained at HQR, Fort Collins, CO, USA, were plated directly onto blood agar (Thermo Scientific). The number of *B. henselae* colonies were counted and calculated as CFU/mL (colony forming units per mL). Six colonies from the two specified time points (days 0 and 9 post infestation) from each experimentally-infected cat were suspended and frozen in PBS for bacterial species and strain identification by 16S-23S ITS PCR amplification and DNA sequencing.

All *Bartonella* sp. culture methods were carried out in a class-3 biosafety cabinet in order to prevent the possibility of specimen contamination and to protect laboratory personnel.

### Molecular Detection of *Bartonella* DNA

#### DNA Extraction

DNA was extracted using the DNeasy^®^ Blood & Tissue Kit (Qiagen^®^, Valencia, California, USA), according to manufacturer’s instructions, from:

- each cat blood sample at pre-innoculation (from seven cats from KSU - used for flea colony maintainance – and from four cats from HQR – two to be experimentally infected with *B. henselae* and two to be used as negative controls);
- each cat blood sample at post- innoculation (four cats from HQR – two experimentally infected with *B. henselae* and two used as negative controls at 5 time points, i.e. one, two, three, four, and six weeks after inoculation);
- cat blood added to liquid growth medium (BAPGM) (C7, C14, and C21) at pre-infection (from seven cats from KSU and from four cats from HQR)
- adult flea pools (up to 10 adult fleas) pre- and –post (after 24 hours and 9 days feeding) flea attachment;
- flea eggs and larvae at pre-inoculation;
- flea dirt at pre- and post- innoculation.
- Before DNA extraction, fleas were pulverized using liquid nitrogen and a sterile pestle (Fisherbrand Pellet Pestle Cordless Motor).

### Quantitative PCR (qPCR) Assay

Quantitative PCR amplification of the *Bartonella* ITS region was performed as described previously (Oteo et al., 2017; Breitschwerdt and Maggi, 2019), with minor modifications, on biological samples obtained from both pre- and post infection period. Oligonucleotide primers BsppITS325s: 5′ CTTCAGATGATG ATCCCAAGCCTTCTGGCG 3′ (Forward) and 543as: 5′ AATTGGTGGGC CTGGGAGGACTTG 3′ (Reverse) and probe BsppITS500probe: 5′ FAM- GTTAGAGCGCGCGCTTGATAAG -IABkFQ 3′ were utilized for these assays. Each 25 μL PCR reaction was prepared using 12.5 μL of SsoAdvanced Universal Probes Supermix (Bio-Rad, Hercules, CA), 0.2 μL of 100 μM of each forward primer, reverse primer, and TaqMan probe (IDT DNA Technology, Coralville, IA), 7.5 μL of Ultra-Pure, molecular grade water (Genesee Scientific, San Diego, CA, USA), and 5 μL of DNA from each sample tested. Quantitative PCR was performed in an CFX96 (Bio-Rad, Hercules, CA) under the following conditions: a single hot-start cycle at 95°C for 3 min followed by 45 cycles of denaturing at 94°C for 10 s, annealing at 68°C for 10 s, and extension at 72°C for 10 s. Amplification was completed by an additional cycle at 72°C for 30 s. Positive amplification was assessed by analysis of detectable fluorescence vs. cycle threshold values (Maggi et al., 2020).

### Droplet Digital PCR (ddPCR) Assay

Droplet digital PCR amplification of the *Bartonella* ITS region was conducted using the same primers and probes employed for qPCR, with minor modifications, on biological samples obtained from pre- infection period. The 22 μL final ddPCR reaction contained 11 μL of ddPCR Supermix for probes (no dUTP) (Bio-Rad, Hercules, CA), 0.2 μL each of 100 μM forward and reverse primers, 0.2 μL of 100 μM probe (IDT DNA Technology, Coralville, IA), 3.8 μL of molecular grade water, 5 μL of DNA from each sample tested, and 1 μL of HindIII DNA restriction enzyme. The ddPCR analysis was performed using a QXONE Droplet Digital PCR (Bio-Rad, Hercules, CA) system under the following conditions: a single hot-start cycle at 95°C for 10 min, followed by 40 cycles of denaturing at 94°C for 30 s, and annealing at 62.9°C for 1 min. A final extension at 98°C was carried out for 5 min. Bio-Rad QuantaSoft Analysis Pro software was utilized to track and analyze the fluorescent drop distribution and positive detection threshold readings for each channel (Maggi et al., 2020).

### Conventional PCR Assays for *Bartonella* spp. Based on ITS and *gltA*

*Bartonella* genus specific 16S-23S ITS-based conventional PCR using forward primer 325s (5′ CTTCAGATGATG ATCCCAAGCCTTCTGGCG 3′) and reverse prime 1100as (5′ GAACCGACGACCCCCTGCTTGCAAAGCA 3′) was performed as described previously (Oteo et al., 2017). In addition, two other set of oligonucleotides, *Bartonella* genus specific *gltA*850 5′ AGAATTCCTGAATTYATTGCACGTGCA 3′ and *gltA*1120 5′ CCTAGAGCTTTTAATGTAATWCCAGAA 3′, were used as forward and reverse primers, respectively, for amplification of a fragmente of the *gltA* gene by conventional PCR. Conventional PCR was performed in a 25 μL final volume reaction containing 12.5 μL of MyTaq HS Red Mix 2× (Bioline), 0.2 μL of 100 μM of each forward and reverse primer (IDT-DNA Technology), 7.3 μL of molecular-grade water, and 5 μL of DNA from each sample tested. Five μL of Ultra-Pure, molecular grade water (Genesee Scientific, San Diego, CA, USA), 5 μL of DNA from the blood of a healthy cat, and 5 μL of DNA extracted from nonincoulated BAPGM-negative controls were used as negative controls. Genomic DNA from *B. henselae* San Antonio Type-2 was used as a positive control. Amplicon products were sequenced by Sanger sequencing using a commercial company (Genewiz, Research Triangle Park, NC). Chromatograms evaluation and sequence alignment was performed using SnapGene software (GSL Biotech; available at snapgene.com) to determine *Bartonella* spp. ITS strain type.

### Biological and Analytical Replicates for Proteomics Workflow

For the proteomics workflow, we generated protein samples from 10 experimental groups, each derived from 325 fleas. Each experimental group was submitted to the proteomic workflow in duplicate. Therefore, each studied condition (unfed fleas; fleas fed on non-infected cats, fleas fed on *Bartonella henselae*-infected cats) was represented by one biological replicate and two analytical replicates. In total, 20 samples from the 5 groups were submitted to the proteomic workflow as follows:

**• Group 1. Unfed fleas**
- Samples 1 and 2 (biological replicate), and respective analytical replicate (1AR, 2 AR)
**• Group 2. Fleas fed on uninfected cats at 24 hours of flea feeding**
- Sample 3: Fleas fed on an uninfected cat (Cat #3508) and respective analytical replicate (3AR)
- Sample 4: Fleas fed on a second uninfected cat (Cat #3363) and respective analytical replicate (4 AR)
**• Group 3. Fleas fed on uninfected cats at 9 days of flea feeding**
- Sample 5: Fleas fed on an uninfected cat (Cat #3508) and respective analytical replicate (5AR)
- Sample 6: Fleas fed on a second uninfected cat (Ca t#3363) (biological replicate) and respective analytical replicate (6 AR)
**• Group 4. Fleas fed on *B. henselae*-infected cats at 24 hours of flea feeding**
- Sample 7: Fleas fed on a *B. henselae*-infected cat (Cat #3320) and respective analytical replicate (7 AR)
- Sample 8: Fleas fed on a second *B. henselae*-infected cat (Cat #3711) (biological replicate) and respective analytical replicate (8 AR)
**• Group 5. Fleas fed on *B. henselae*-infected cats at 9 days of flea feeding**
- Sample 9: Fleas fed on a *B. henselae*-infected cat (Cat #3320) and respective analytical replicate (9 AR)
- Sample 10: Fleas fed on a second *B. henselae*-infected cat (Cat #3711) (biological replicate) and respective analytical replicate (10 AR)

### Protein Extraction

Three hundred twenty fleas from each experimental group were initially pulverized in liquid nitrogen and mixed with 4 mL buffer (0.25 M sucrose, 1 mM MgCl_2_, 10 mM Tris-HCl, pH 7.4) supplemented with 4% SDS and Halt protease inhibitor cocktail (Thermo Scientific). Each sample was homogenized in a bead-beating instrument (Fisherbrand) with disruption glass beads (1mm) (Research Products International Corp.) according to the following the conditions: 1 cycle at 5 m/s for 40 s; 1 cycle at 5 m/s for 30 s; 1 cycle at 5 m/s for 20 s; 1 cycle at 5 m/s for 10 s. Samples were sonicated for 1 min in an ultrasonic cooled bath followed by 10 sec of vortexing. After 3 cycles of sonication-vortexing, the homogenate was centrifuged at 206 g for 5 min at room temperature to remove cellular debris. The supernatant was collected and protein concentration was determined using the BCA Protein Assay (Thermo Scientific, San Jose, CA, USA) using BSA as a standard (Contreras et al., 2018, with modifications).

### Filter Aided Sample Preparation (FASP)

The extracted flea proteins samples were thawed and subjected to an acidified (100 mM HCl) acetone-based precipitation to cleanse the constituent protein. A Nanodrop A280 (Thermo Scientific, Wilmington, DE) was used for quantification of total protein in each sample. Two separate volume equivalents of 200 µg (per sample or biological replicate), representing two distinct analytical replicates, were lyophilized in separate microcentrifuge tubes and subsequently taken through a modified filter-aided sample preparation (FASP) protocol (Wiśniewski et al., 2009; Pérez-Rodriguez et al., 2020). Briefly, 200 μg of protein from a given sample was dissolved, with vortexing, in 200 μL of 50 mM ammonium bicarbonate (ABC) with 1% sodium deoxycholate (SDC) to achieve a final protein concentration of 1 µg/µL. For reduction of protein disulfide bonds, 15 μL of 50 mM dithiothreitol (DTT) in 0.1 M Tris-HCl pH 8 was added to the protein to arrive at a final DDT concentration of ~3.5 mM. The samples were incubated for 30 min at 56°C and then allowed to cool to room temperature (~ 5 min) before proceeding to the next step. A 200 µL volume of 8 M urea in 0.1M TRIS-HCl pH 8 was then added to each sample, with thorough vortexing to aid in protein denaturation. Each sample solution was then transferred onto separate Vivacon 500 10 kDa molecular weight cutoff (MWCO) filtration units (Sartorius Stedim Biotech, Goettingen, Germany). The samples were then centrifuged at 12,500 x *g* for 15 min. An extra 200 µL of 8 M urea in 0.1M TRIS-HCl pH 8 was added to each sample filter (no centrifugation step). For alkylation of free sulfur groups, 64 μL of 200 mM iodoacetamide (IAA, prepared in 8 M urea containing 0.1M TRIS-HCl pH 8) was added to the 8 M urea solution already present in each sample filter, to reach a final concentration of ~50 mM IAA. The reaction was allowed to proceed in the dark at room temperature for 1 h, followed by centrifugation at 12,500 × *g* for 25 min. Reduction and alkylation of the sample protein was followed by a series of washing steps in preparation for proteolytic digestion. A 100 µL volume of 2 M urea with 10 mM CaCl_2_ in 0.1M Tris-HCl pH 8 was then added to each filter and the filtration units were centrifuged at 12,500 × *g* for 15 min. This step was repeated two more times to ensure a thorough wash. The flow-through was discarded from the collection tubes following these washing steps. A 100 µL volume of 0.1M TRIS-HCl pH 7.5 was then added to each filter and the filtration units were centrifuged at 12,500 × *g* for 15 min. This washing step was also repeated two more times and the flow-through solutions were discarded. After replacing the collection tubes with fresh ones, 100 μL of 0.1M TRIS-HCl pH 7.5 was added to each filter as the digestion buffer. Next, 100 µL Trypsin Gold (Promega, Madison, WI) at a concentration of 0.04 µg/µL was added to each filter. This yielded a substrate: enzyme ratio of 50:1. After overnight digestion at 37°C, 50 µL of quench buffer (0.001% Zwitttergent 3-16 in 1% formic acid) was added to each sample filter and the filtration units were centrifuged at 12,500 x *g* for 15 min. The process was repeated with 400 µL of quench buffer and centrifugation at 13,000 x *g* for 30 min to recover the maximum amount of tryptic peptides. The filters were discarded and the flow-through for each sample (containing peptide digests) was retained, lyophilized and stored at -20°C until analysis by nanoLC-MS/MS (Wiśniewski et al., 2009).

### LC-MS/MS Analysis

The protein digests were reconstituted with 200 µL Mobile Phase A (MPA, 2% acetonitrile in water with 0.1% formic acid) and 2 µL injections were analyzed by reversed phase nano - liquid chromatography (LC) - mass spectrometry (MS) (nano-LC-MS/MS) using an Easy-Nano-1200 nanoLC system (Thermo Scientific, San Jose, CA, USA) interfaced with an Orbitrap Exploris 480 (Thermo Scientific) Mass Spectrometer. The sample peptides were concentrated, desalted and separated using a ‘trap and elute’ column configuration consisting of a 0.075 mm × 20 mm C_18_ trap column with particle size of 3 µm (Thermo Scientific Accclaim PepMap™ 100, Part # 164946) in line with a 0.075 mm × 250 mm C_18_ nanoLC analytical column with particle size of 2 µm (Thermo Scientific PepMap™, Part # ES902). The flowrate was maintained at 300 nL/min. Peptides were eluted using a 135 min solvent gradient, ramping from 5 to 25% Mobile Phase B (MPB, 80% acetonitrile with 0.1% formic acid) over 102 min, followed by another ramp to 40% MPB over 15 min, and then a steep ramp to 95% MPB in 1 min, at which point MPB was maintained at 95% for 17 min for column washing. Eluting tryptic peptides were ionizaed by subjecting them to 19 kV in the ion source for electrospray ionization. The ion trasnfer tube temperature was maintained at 175°C. The peptides were interrogated by full MS scan and data dependent acquisition (DDA) MS/MS. A cycle time of 1.5 s was employed between master scans. Full MS data was collected with a *m/z* scan range of 375 to 1,600 in positive ion mode at 120 K resolving power with 300% normalized AGC Target, 120 ms maximum injection time and RF lens of 40%. MS/MS scans were collected with at 15 K mass resolving power, with 1.5 *m/z* isolation window, 30% normalized HCD collision energy, 100% normalized AGC Target, 21 ms maximum injection time and dynamic exclusion applied for 20 s periods.

### Data Interrogation

The raw nanoLC-MS/MS files were interrogated with Proteome Discoverer 2.4.0.308 (PD, Thermo Scientific, San Jose, CA) software against the Siphonaptera (2,079 sequences), *Ctenocephalides felis* (412 sequences)*, Felis catus* (50,040 sequences), and *Bartonella henselae* (4111 sequences) Uniprot protein databases (downloaded 10/04/2020). The data was also searched against a contaminants database (69 sequences) to identify potential contaminants during the experiments. These databases were searched with the following parameters: trypsin (full) as the digesting enzyme, a maximum of 3 missed trypsin cleavage sites allowed, 5 ppm precursor mass tolerance, 0.02 Da fragment mass tolerance, dynamic modifications on [a] methionine (oxidation) [b] asparagine (deamidated) [c] glutamine (deamidated), as well as static carbamidomethyl modifications on cysteine residues. The SEQUEST HT algorithm was employed in data interrogation (Tabb, 2015). This algorithm compares the observed peptide MS/MS spectra and theoretically derived spectra from the database to assign quality scores. These quality scores and other important predictors are combined in the algorithm, which assigns an overall score to each peptide. The confidence in protein identification is increased with the number of distinct amino acid sequences identified. Therefore, proteins are normally categorized into a different priority group depending on whether they have only one or multiple unique sequences of the required peptide identification confidence. Percolator peptide validation was based on the *q*-value (adjusted p-value) and minimal false discovery rate (FDR) < 0.01 was considered as a condition for successful peptide assignments.

### Estimation of Protein Relative Abundances and Statistical Analysis

Peptide-spectrum matches for each identified protein were normalized according to the NSAF criteria (Paoletti et al., 2006). When necessary, missing value imputation was carried out by using the minimum NSAF value of the biological sample. Statistical analyses were performed in Inferno RDN 1.1.7. Pairwise comparisons were carried out to identify changes in the abundance of proteins according to the Mann-Whitney U-test. The identifications with a p-value <0.05 and, at the same time, a fold-change ≥ 2 were defined as significantly abundant proteins. For heat map construction, NSAF values of all protein identifications in the Siphonaptera and *Ctenocephalides felis* databases were log_2_ transformed. Hierarchical clustering was performed for flea protein identifications using Euclidean distance and the Perseus (v 1.6.14.0) default program parameters (Tyanova et al., 2016).

## Results

### Screening of Cats, Fleas, Flea Dirt, Flea Eggs and Larvae for *Bartonella* spp. (Pre-Inoculation Period)

Blood samples from seven cats from KSU (used for flea colony maintenance) and from four cats from HQR, Fort Collins, CO, USA (prior to challenge with *Bh* CSU-1 or saline) were qPCR and ddPCR negative for *Bartonella* spp. DNA based on ITS region amplification and were also qPCR/ddPCR negative by BAPGM enrichment blood culture at 7, 14, and 21 days following blood inoculation. Direct plating of blood samples and BAPGM cultures from these 11 cats onto blood agar plates resulted in no growth of microorganisms. Similarly, a batch of flea eggs, larvae, adult (unfed) fleas and flea dirt from KSU similarly tested by qPCR and ddPCR were negative for *Bartonella* spp. DNA targeting the 16S-23S ITS region.

### Experimental Infection of Cats

Two cats (#3320 and #3711) were experimentally infected with the CSU Bh-1 strain. Seven days after the infection, bacteremia was documented in both cats by blood qPCR testing. Based on blood culture and *Bartonella* ITS-qPCR, both cats were again *B. henselae* bacteremic at the end of the experiment (day 9 of flea infestation). Based upon IFAT IgG antibody titers, both cats seroconverted: cat #3320 titers increased from 1:128 to 1:1024, cat #3711 titers increased from 1:32 to 1:512. Pools of 15 adult fed fleas as well flea dirt collected from the chambers from each *B. henselae-*inoculated cat at the two selected time points (24 hours and 9 days of flea infestation, respectively) were also *Bartonella* spp. ITS qPCR positive. This part of the experiment confirmed that fleas recovered from cats #3320 and #3711 had ingested *B. henselae*-containing blood, based upon *B. henselae* IgG seroconversion, isolation of *B. henselae* colonies onto blood agar plates, and the amplification of *Bartonella* DNA from each cat’s blood and from BAPGM liquid enrichment blood cultures (C7, C14, and C21 day cultures at 24 hours and 9 days after flea infestation). Also, *Bartonella* DNA was detected in adult fleas that fed on each cat as well as in flea dirt excreted by the fleas following feeding. At the time of flea infestation, i.e. four weeks after the experimental infection of cats with Bh CSU-1 strain, the bacteremia level was 2.4 x 10^4^ CFU/mL and 1.4 x 10^5^ CFU/mL for cats #3320 and #3711, respectively. Nine days after the initial flea infestation, when the remaining flea-chambers were removed from the cats, the *B. henselae* bacteremia was 2 x 10^3^ CFU/mL and 5.8 x 10^4^ CFU/mL for cats #3320 and #3711, respectively. Six *Bartonella* colonies obtained from blood agar plates inoculated with blood samples from each experimentally cat at the two study time points were analyzed by ITS-based conventional PCR followed by DNA sequencing. All *Bartonella* ITS sequences obtained from 24 colonies were 100% identical to each other and a representative sequence was deposited in the Genbank database under accession number OK275541.

The two cats (#3363 and #3508) inoculated with saline solution (negative controls) remained negative by *Bartonella* ITS-based qPCR, routine blood culture, BAPGM enrichment cultures negative throughout the experiment and did not develop antibodies based on IFAT results. Pools of 15 adult fed fleas as well flea dirt collected from the chambers from each control cat at 24 hours and 9 days following flea infestation, were also *Bartonella* spp. ITS qPCR negative. Based upon the lack of *B. henselae* IgG seroconversion, failure to isolate *B. henselae* colonies onto blood agar plates, the lack of amplification of *Bartonella* DNA from each cat’s blood and BAPGM liquid enrichment blood cultures (C7, C14, and C21 days), at the 24 hr and 9 day timepoints after flea infestation, from adult fleas that fed on each cat, and from flea dirt excreted by the fleas following feeding, we confirmed that the fleas recovered from cats #3363 and #3508 had ingested *Bartonella-*free blood.

The detailed information on serology, qPCR, and culture results for each of the four cats and associated fleas are summarized in **Supplementary Material Tables 1–4 (SM 1-4)**. Three hundred forty fleas were recovered at each time point (24 hours and 9 days) after infestation.

### Protein Concentration Assessment

The protein concentration of each pool of fleas (n=320) belonging to each experimental group is presented in **Supplementary Material Table SM5**.

### Proteomic Data Availability

The data has been uploaded to the following link: https://panoramaweb.org/NCSU%20-%20METRIC/METRIC%20Public%20Data/20210320%E2%80%94%E2%80%94CatFleaBartanella/project-begin.view.

### Effect of the Duration of (Time-Dependent) Blood Feeding on *C. felis* FDAP

Unfed fleas had the following overrepresented FDAP when compared to fleas fed on uninfected cats for 24 hours: Serpin 1, Peritrophin-like protein 1, Arginine kinase 2, and Trypsin. Additionally, unfed fleas also had Succinate-semialdehyde dehydrogenase, Arginine kinases 1 and 2, Peritrophin-like protein 1 and 2, FS-H/FSI antigen family member 3, and Trypsin and Chymotrypsin-like serine proteases as overrepresented FDAP when compared to fleas fed on uninfected cats for 9 days. Serine proteases showed the highest fold changes (3.82 and 4.72 in log_2_ scale) in unfed fleas when compared to fleas fed on uninfected cats (**Table 1**).

**Table 1:** Uniprot identification number, associated protein, reference organism, cellular localization, biological process and molecular function of FDAP (Flea Differentially Abundant Proteins) in unfed fleas when compared to fleas fed on uninfected cats for 24 hours and 9 days.

Uniprot ID	Protein	Organism	Localization	Biological Process	Molecular Function	Fleas fed on uninfected cats for 24 hours*	Fleas fed on uninfected cats for 9 days*
Q8IS47	Serpin 1	*C. felis*	Extracellular space	negative regulation of peptidase activity	Serine-protease inhibitor	1.28	–
Q8N0M9	Peritrophin-like protein 1	*C. felis*	Extracellular space	Metabolic process	Chitin-biding	1.6	1.84
Q8N0M8	Peritrophin-like protein 2					–	3.37
C6H0K6	Arginine kinase 2	*C. felis*		Phosphocreatine biosynthetic process; phosphorylation	Catalytic process; nucleotide binding; ATP binding arginine kinase activity; transferase activity, transferring phosphorus-containing groups	2.34	1.29
C6H0K8	Arginine kinase 1					–	1.16
D0V532	Trypsin	*C. felis*		proteolysis	Hydrolyse activity; serine-type peptidase activity	**4.71**	3.4
Q9XY63	Trypsin-like serine protease					–	1.81
Q9XY53	Chymotrypsin-like serine protease					–	**3.82**
B0JFD7	Succinate-semialdehyde dehydrogenase	*C. felis*	mitochondrion	gamma-aminobutyric acid catabolic process	oxidoreductase activity, acting on the aldehyde or oxo group of donors, NAD or NADP as aceptor; succinate-semialdehyde dehydrogenase (NAD(P)+) activity	–	1.0
I3VPE4	FS-H/FSI antigen family member 3	*C. felis*				–	3.4

^*^Pairwise comparisons were carried out to identify changes in the abundance of proteins according to the Mann-Whitney U-test. The identifications with a p-value <0.05 and, at the same time, a fold-change ≥ 2 were defined as significantly abundant proteins. Fold changes are presented in log_2_ scale. The highest fold changes for each pairwise comparison are bolded.

Regarding the response of cat fleas to blood feeding, the FDAP of fleas fed on uninfected cats for 24 hours when compared to unfed fleas were Trypsin/Chymotrypsin like serine proteases, ribosomal proteins, Vitellogenin C, Proteasome subunit alpha type, Glycinamide ribotide transformylase, Allantoinase, and Juvenile hormone epoxide hydrolase 1 (**Table 2**). Alternatively, the FDAP of fleas fed on uninfected cats for 9 days when compared to unfed fleas were the same as mentioned above (except for Proteasome subunit alpha type and Juvenile hormone epoxide hydrolase 1) plus Elongation factor 1-alpha (**Table 3**). While Serine proteases had the highest fold change (5.7 in log_2_ scale) in fleas fed on uninfected cats for 24 hours when compared to unfed fleas (**Table 2**), Vitellogenin had the highest fold change (5.7 in log_2_ scale) in fleas fed on uninfected cats for 9 days when compared to unfed fleas (**Table 3**).

**Table 2:** Uniprot identification number, associated protein, reference organism, cellular localization, biological process and molecular function of FDAP (Flea Differentially Abundant Proteins) in fleas fed on uninfected cats for 24 hours when compared to unfed fleas.

Uniprot ID	Protein	Organism	Localization	Biological Process	Molecular Function	Unfed Fleas*
Q9XY55	Trypsin-like serine protease	*C. felis*		proteolysis	serine-type endopeptidase activity; hydrolase activity	**5.7**
D0V531						4.5
Q9XY51						2.577
Q9XY59						1.518
Q9XY47	Chymotrypsin-like serine protease	*C. felis*		proteolysis	serine-type endopeptidase activity; hydrolase activity; peptidase activity	4.9
A0A0K0TN27						4.2
D0EL77						3.3
D0V543						1.972
Q9XY45						1.931
D0V544						1.761
I3VPD2	Vitellogenin C fragment	*C. felis*				4.8331
A2IAD8	Ribosomal protein S11-2	*X. cheopis*	ribosome	translation	structural constituent of ribosome nucleic acid binding	3.6
I3VPD7	60S ribosomal protein L13a-like protein					2.93
A2IAE0	Ribosomal protein S18					3.5
I3VPG1	60S ribosomal protein	*C. felis*				4.8333
I3VPC1	60S ribosomal protein L32					2.96
I3VPD0	Ribosomal protein L44e (Fragment)					2.840
I3VPC3	40S ribosomal protein S6					2.798
I3VPD6	Ribosomal protein S4					2.787
I3VPC8	Ribosomal protein L35Ae (Fragment)					2.449
I3VPC7	Ribosomal protein L23e (Fragment)					2.240
I3VPB8	60S ribosomal protein L8 (Fragment)					2.192
I3VPB9	40S ribosomal protein S16 (Fragment)					2.114
I3VPC2	60S ribosomal protein L18a (Fragment)					2.031
A2IAA3	Proteasome subunit alpha type	*X. cheopis*	Nucleus; cytoplasm; proteasome core complex, alpha-subunit complex	ubiquitin-dependent protein catabolic process		2.889
K7ZI81	Glycinamide ribotide transformylase (Fragment)	*C. felis*		‘*de novo*’ IMP biosynthetic process	ATP binding; phosphoribosylformylglycinamidine cyclo-ligase activity; ligase activity; nucleotide binding	2.716
Q8I6V5	Allantoinase	*C. felis*		Allantoin catabolic process	allantoinase activity; zinc ion binding; hydrolase activity; hydrolase activity, acting on carbon-nitrogen (but not peptide) bonds, in cyclic amides; cobalt ion binding; metal ion binding	2.427
Q8MZR6	Juvenile hormone epoxide hydrolase 1	*C. felis*	integral component of membrane; membrane; endoplasmic reticulum; intracellular membrane-bounded organelle; endoplasmic reticulum membrane	Aromatic compound catabolic process	catalytic activity ; cis-stilbene-oxide hydrolase activity; hydrolase activity	2.05

^*^Pairwise comparisons were carried out to identify changes in the abundance of proteins according to the Mann-Whitney U-test. The identifications with a p-value <0.05 and, at the same time, a fold-change ≥ 2 were defined as significantly abundant proteins. Fold changes are presented in log_2_ scale. The highest fold changes for each pairwise comparison are bolded.

**Table 3:** Uniprot identification number, associated protein, reference organism, cellular localization, biological process and molecular function of FDAP (Flea Differentially Abundant Proteins) in fleas fed on uninfected cats for 9 days when compared to unfed fleas.

Uniprot ID	Protein	Organism	Localization	Biological Process	Molecular Function	Unfed Fleas*
I3VPD2	Vitellogenin C (Fragment)	*C. felis*				**5.718**
Q9XY47	Chymotrypsin-like serine protease	*C. felis*		proteolysis	serine-type endopeptidase activity; hydrolase activity; peptidase activity	5.439
A0A0K0TN27						4.393
Q9XY54						4.001
Q9XY49						3.921
D0EL77						3.610
Q9XY45						2.096
D0V543						1.748
D0V544						1.633
Q9XY55	Trypsin-like serine protease					4.926
D0V531						3.839
Q9XY51						1.728
Q9XY59						1.467
A0A0G2UNM7	Elongation factor 1-alpha (Fragment)	*Craneopsylla minerva wolffheuglia*		Translational elongation; Translational elongation factor activity	GTPase activity; GTP binding;	4.738
A0A0G2UW18		*Paramonopsyllus scalonae*	cytoplasm			3.681
A0A0G2UVZ3		*Synopsyllus girardi*				3.600
I3VPG1	60S ribosomal protein L26	*C. felis*	ribosome	translation	structural constituent of ribosome; nucleic acid binding	2.494
K7ZI81	Glycinamide ribotide transformylase (Fragment)	*C. felis*		‘*de novo*’ IMP biosynthetic process	ATP binding; phosphoribosylformylglycinamidine cyclo-ligase activity; ligase activity; nucleotide binding	2.311
Q8I6V5	Allantoinase	*C. felis*		Allantoin catabolic process	allantoinase activity; zinc ion binding; hydrolase activity; hydrolase activity, acting on carbon-nitrogen (but not peptide) bonds, in cyclic amides; cobalt ion binding; metal ion binding	1.950

*Pairwise comparisons were carried out to identify changes in the abundance of proteins according to the Mann-Whitney U-test. The identifications with a p-value <0.05 and, at the same time, a fold-change ≥ 2 were defined as significantly abundant proteins. Fold changes are presented in log2 scale. The highest fold change is bolded.

When time-dependent blood feeding was considered, the FDAP of fleas fed on uninfected cats between 24 hours and 9 days included elongation factor 1-alpha, ribosomal proteins, Proteasome subunits beta and alpha, and Juvenile hormone epoxide hydrolase 1. An increase in elongation-factor 1 alpha had the highest fold change (5.692 in log_2_ scale) between 24 hours and 9 days in fleas fed on the uninfected control cats (**Table 4**).

**Table 4:** Uniprot identification number, associated protein, and reference organism of FDAP (Flea Differentially Abundant Proteins) in fleas fed on uninfected cats for 24 hours when compared to fleas fed on uninfected cats for 9 days.

Uniprot ID	Protein	Organism	Fleas fed on uninfected cats for 9 days*
Q8MWJ4	Elongation factor 1-alpha (Fragment)	*Panorpa debilis*	**5.692**
I3VPG1	60S ribosomal protein L26	*C. felis*	3.4
I3VPB8	60S ribosomal protein L8 (Fragment)	*C. felis*	2.308
I3VPD6	Ribosomal protein S4	*C. felis*	1.992
A2IAA2	Proteasome subunit beta	*X. cheopis*	1.814
A2IAD8	Ribosomal protein S11-2	*X. cheopis*	1.614
Q8MZR6	Juvenile hormone epoxide hydrolase 1	*C. felis*	1.581
A2IAA3	Proteasome subunit alpha type	*X. cheopis*	1.402

*Pairwise comparisons were carried out to identify changes in the abundance of proteins according to the Mann-Whitney U-test. The identifications with a p-value <0.05 and, at the same time, a fold-change ≥ 2 were defined as significantly abundant proteins. Fold changes are presented in log_2_ scale. The highest fold change is bolded.

### Effect of *B. henselae* Infection on *C. felis* FDAP

When comparing the differentially abundant proteins for fleas fed on uninfected cats for 24 hours to fleas fed on *B. henselae*-infected cats for 24 hours serine protease abundance was increased in the former group. On the other hand, the FDAP of fleas fed on uninfected cats for 9 days documented increased abundance of Elongation factor 1-alpha and Serine proteases when compared to fleas fed on *B. henselae*-infected cats for 9 days (**Table 5**).

**Table 5:** Uniprot identification number, associated protein, and reference organism of FDAP (Flea Differentially Abundant Proteins) in fleas fed on uninfected cats for 24 hours when compared to fleas fed on *B. henselae*-infected cats for 24 hours, and fleas fed on uninfected cats for 9 days when compared to fleas fed on *B. henselae*-infected cats for 9 days.

Uniprot ID	Protein	Organism	Fleas fed on uninfected cats for 24 hours vs. fleas fed on *B. henselae*-infected cats for 24 hours*	Fleas fed on uninfected cats for 9 days vs. fleas fed on *B. henselae*-infected cats for 9 days*
Q9XY52	Trypsin-like serine protease (Fragment)	*C. felis*	**3.387**	–
A0A0K0TN27	Chymotrypsin-like serine protease	*C. felis*	1.518	–
D0V533	Trypsin (Fragment)	*C. felis*	1.217	–
Q8MWN7	Elongation factor 1-alpha (Fragment)	*Papilio troilus*	–	**3.613**
B6CNA9	Elongation factor 1-alpha (Fragment)	*Corypsylla ornata*	–	3.587
Q9XY52	Trypsin-like serine protease (Fragment)	*C. felis*	–	2.531

*Pairwise comparisons were carried out to identify changes in the abundance of proteins according to the Mann-Whitney U-test. The identifications with a p-value <0.05 and, at the same time, a fold-change ≥ 2 were defined as significantly abundant proteins. Fold changes are presented in log_2_ scale. The highest fold changes for each pairwise comparison are bolded.

When fleas fed on *B. henselae*-infected cats for 9 days were compared to fleas fed on uninfected cats for 9 days, Succinate-semialdehyde dehydrogenase, Phosphoenolpyruvate carboxykinase, and 40S ribosomal protein S7 had higher abundance levels (**Table 6**). Succinate-semialdehyde dehydrogenase was the protein with the highest fold change when these two groups were compared.

**Table 6:** Uniprot identification number, associated protein, reference organism, cellular localization, biological process and molecular function of FDAP (Flea Differentially Abundant Proteins) in fleas fed on *B. henselae*-infected cats for 9 days when compared to fleas fed on uninfected cats for 9 days.

Uniprot ID	Protein	Organism	Localization	Biological Process	Molecular Function	Fleas fed on *B. henselae*-infected cats vs. fleas fed on uninfected cats for 9 days*
B0JFD7	Succinate-semialdehyde dehydrogenase	*C. felis*	mitochondrion	gamma-aminobutyric acid catabolic process	Succinate-semialdehyde dehydrogenase (NAD(P+) activity; oxireductase activity, acting on the aldehyde or oxo group of donors,	**1.620**
Q27544	Phosphoenolpyruvate carboxykinase	*C. felis*		Gluconeogenesis; phosphorylation	Lyase activity; GTP binding; purine nucleotide binding; phosphoenolpyruvate carboxykinase (GTP) activity; kinase activity	1.208
I3VPD4	40S ribosomal protein S7 (Fragment)	*C. felis*	ribosome	translation	Structural constituent of ribosome	1.032

*Pairwise comparisons were carried out to identify changes in the abundance of proteins according to the Mann-Whitney U-test. The identifications with a p-value <0.05 and, at the same time, a fold-change ≥ 2 were defined as significantly abundant proteins. Fold changes are presented in log_2_ scale. The highest fold change is bolded.

When fleas fed on *B. henselae*-infected cats were compared, taking into account the two selected time points (24 hours and 9 days), elongation factor-1 alpha and ribosomal protein were the most abundant proteins among fleas fed on *B. henselae* infected cats for 24 hours when compared to fleas fed on *B. henselae* infected cats for 9 days **(** **Table 7** **)**. In contrast, serine proteases, secreted salivary acid phosphatase, and ribosomal protein were identified in higher levels in fleas fed on *B. henselae* infected cats for 9 days when compared to those fed for 24 hours. While Elongation factor 1-alpha was the DAP with the highest fold change in fleas fed on *B. henselae* infected cats for 24 hours when compared to those fed for 9 days, Chymotrypsin-like serine protease had the highest fold change when fleas fed on *B. henselae* infected cats for 9 days were compared to those fed for 24 hours.

**Table 7:** Uniprot identification number, associated protein, and reference organism of FDAP (Flea Differentially Abundant Proteins) in fleas fed on *B. henselae*-infected cats for 24 hours when compared to those fed for 9 days, and fleas fed on *B. henselae*-infected cats for 9 days when compared to those fed for 24 hours.

Uniprot ID	Protein	Organism	Fleas fed on *B. henselae*-infected cats for 24 hours vs. 9 days*	Fleas fed on *B. henselae*-infected cats for 9 days vs. 24 hours*
B6CNA1	Elongation factor 1-alpha (Fragment)	*Ornithophaga anomala anomala*	**3.468**	–
A2IAD8	Ribosomal protein S11-2	*X. cheopis*	2.598	–
A0A0G2UVZ8	Elongation factor 1-alpha (Fragment)	*Wenzella obscura*	2.442	–
B6CND3	Elongation factor 1-alpha (Fragment)	*Citellophilus sparsilis*	2.227	**-**
Q9XY54	Chymotrypsin-like serine protease	*C. felis*		**3.534**
I3VPB2	Secreted salivary acid phosphatase (Fragment)	*C. felis*		3.05
Q9XY62	Chymotrypsin-like serine protease	*C. felis*		2.722
Q9XY49	Chymotrypsin-like serine protease	*C. felis*		1.96
B0JFD7	Succinate-semialdehyde dehydrogenase	*C. felis*		1.695
I3VPF5	40S ribosomal protein S21	*C. felis*		1.473
Q9XY47	Chymotrypsin-like serine protease	*C. felis*		1.326
Q9XY45	Chymotrypsin-like serine protease	*C. felis*		1.197

*Pairwise comparisons were carried out to identify changes in the abundance of proteins according to the Mann-Whitney U-test. The identifications with a p-value <0.05 and, at the same time, a fold-change ≥ 2 were defined as significantly abundant proteins. Fold changes are presented in log_2_ scale. The highest fold changes for each pairwise comparison are bolded.

**Figure 3:**
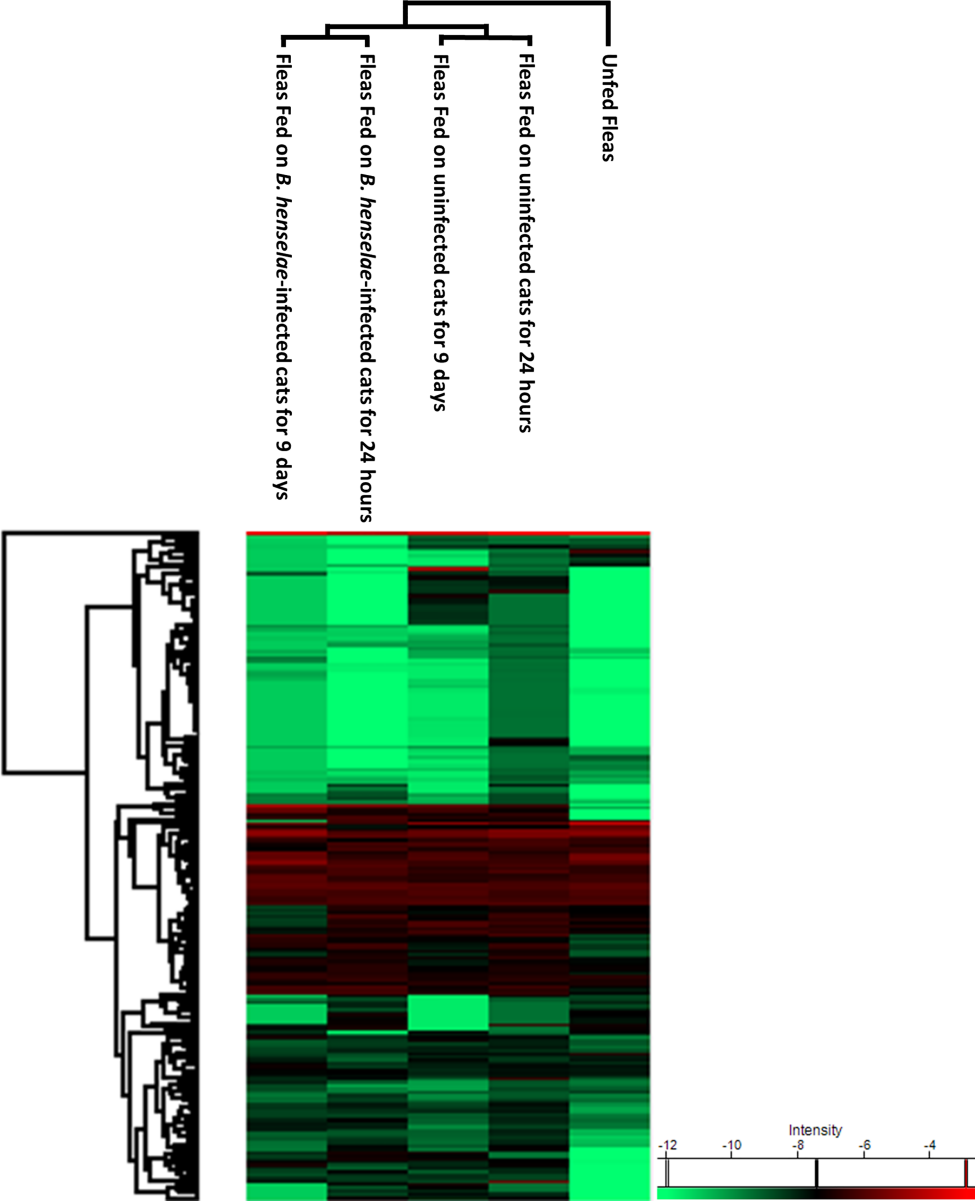
Heat map of all identified proteins in the present study (unfed fleas, fleas fed on uninfected cats for 24 hours and 9 days, and fleas fed on *B. henselae*-infected cats for 24 hours and 9 days).

The protein abundance levels in unfed fleas and in the four study groups are represented in a heat map (**Figure 3**).

## Discussion

The goal of this study was to identify and characterize the proteomic response of the cat flea, *C. felis*, to feeding of uninfected cat blood and blood from cats experimentally infected with *B. henselae*, the causative agent of Cat Scratch Disease and other pathologies reported in animals and human patients. In this study, fleas were actively fed on *B. henselae-*infected cats rather than feeding *C. felis* on *B. henselae-*infected blood in an artificial feeding apparatus, mimicking a more natural feeding model. This flea is generally considered the most prevalent and efficient vector for *B. henselae* (Chomel et al., 1996). The response of *C. felis* and *X. cheopis* to blood feeding by transcriptomic analysis (Dreher-Lesnick et al., 2010; Ribeiro et al., 2012; Greene et al., 2015; Bland et al., 2020) or proteomic approaches (Contreras et al., 2018) has already been reported; however, this is the first study to investigate the response of *C. felis* when ingesting uninfected blood compared to *B. henselae* infected blood. The response of fleas to ingestion of selected pathogens has been investigated in the last decade by transcriptomic approaches, in an attempt to understand the crosstalk between siphonapterans and various bacterial pathogens of medical and veterinary importance. For instance, the responses of *X. cheopis*’ digestive tract to infection by *Y. pestis* (Bland et al., 2020), *C. felis’* midgut to *Rickettsia felis* (Dreher-Lesnick et al., 2010), and *C. felis’* salivary glands to *R. typhi* (Danchenko et al., 2021) have been previously reported. Herein, we used proteomic approaches to investigate if: i.) FDAP (flea differentially abundant proteins) are blood feeding or time-dependent; ii.) and whether *B. henselae* influences flea proteomic changes in a fed versus nonfed state or in a time dependent manner after obtaining a blood meal. The following subtitles of this section discuss the most abundant proteins expressed in cat fleas in response to feeding on uninfected cats and *B. henselae* bacteremic cats at two pre-selected time points (24 hours and 9 days).

### Serine Proteases

Herein, serine proteases had the highest fold changes in the majority of pairwise comparisons. Similar to several other blood-sucking insects, *C. felis* has a plethora of serine proteases (Gaines et al., 1999), which play a role in numerous biological processes, including but not restricted to zymogen activation and digestion. Indeed, serine proteases (trypsin and chymotrypsin) represent the most abundant digestive enzymes in fleas (Gaines et al., 1999; Dreher-Lesnick et al., 2010; Greene et al., 2015; Brown, 2019; Bland et al., 2020). These proteases convert proteins into smaller peptides and represent the most common digestive enzymes in blood-feeding arthropods (Bland et al., 2020). Previously, Greene et al. (2015) identified two serine protease-associated genes (chymotrypsin-like serine protease) in *C. felis* after 24 hours-blood feeding.

Although trypsin and chymotrypsin are primarily considered blood feeding-associated molecules, the presence of ingested pathogens seems to modulate their abundance (Brown, 2019). In our study, serine proteases were associated with the *B. henselae* infection status of *C. felis*, suggesting an additional role in the flea immune response, as previously reported (Dreher-Lesnick et al., 2010; Brown, 2019; Bland et al., 2020). Based on cDNA library expression from *Xenopsylla cheopis* whole flea tissues, a putative chymotrypsin 2-like serine protease was significantly upregulated in response to infection with *Yersinia pestis*, the etiological agent of plague. Similarly, the analysis of *C. felis* cDNA libraries from blood-fed and *Rickettsia typhi*-infected midguts showed differential expression patterns for trypsins and chymotrypsins, with some transcripts exclusively expressed in response to rickettsial infection (Dreher-Lesnick et al., 2010). It is unclear if digestive proteases may help to limit pathogen infection of fleas (Brown, 2019). Our results corroborate previous studies by documenting high fold changes in the abundance of serine proteins in response to blood feeding and concurrent *B. henselae* infection.

### Serpin-1

Serpin-1 had higher abundance levels in unfed fleas when compared to fleas fed on uninfected cats for 24 hours. Serpin-1 belongs to the ubiquitous serine protease inhibitor (serpin) superfamily, which plays important roles in the regulation of several physiological processes by controlling the activity of proteases (Gubb et al., 2010). Indeed, multiple serpins have already been identified in *C. felis* (Brandt et al., 2004). Previously, Brandt et al. (2004) found a strong immunostaining of Serpin-2 in the cells lining the midgut and proventriculus of unfed male *C. felis*, corroborating the high abundance levels of Serpin-1 in unfed fleas observed in the present study. Thus, flea serpins are present and active prior to initiation of blood feeding, potentially to prevent deleterious serine protease activity, by directly inhibiting active serine proteases or by inhibiting the activation of serine protease pro-enzymes. Also, Serpins also have a role after ingestion of a blood meal, presumably to protect the flea gut from extensive proteolytic activity (Greene et al., 2015). For instance, three serpin genes (Serpin 4, 5 and 7) were upregulated in *C. felis* in response to feeding (Dreher-Lesnick et al., 2010). Greene et al. (2015) demonstrated, by suppression subtractive hybridization, an increase of Serpin 3 expression in both *C. felis*’ midgut and carcass in response to 24 hours feeding. In our study serpins did not have higher levels in fed fleas when compared to unfed fleas. Besides midgut and carcass, serpins were immunostained in fed female *C. felis*’ fat body, pericardial cells and in rectal pads, suggesting a role in processes other than blood digestion. Serpins have been implicated in several processes in insects, including development, reproduction (egg development), longevity, innate immunity, tissue remodeling, and inhibition of activation of blood clotting (Brandt et al., 2004). Even though serpins were not upregulated in *B. henselae*-infected fleas in the present study, these proteins have been incriminated in the modulation of flea gut defense mechanisms, by enhancing or suppressing pathogen survival in the flea gut (Brown, 2019).

### Arginine Kinase

Arginine kinase (AK) had higher abundance levels in unfed fleas when compared to fleas fed on uninfected cats for 24 hours and 9 days. AK is the only member of the phosphagen kinase family that has thus far been identified in insects, playing a key role in cellular energy metabolism. In fleas, phospho-L-arginine/AK system may also contribute to a temporal energy buffer (Werr et al., 2009). To date, the role of AK in insects has not yet been clearly defined and is likely to be distinct in different insects, organs and developmental stages (Werr et al., 2009). Previously, two arginine kinase genes were identified in *C. felis*, namely *cfak1* and *cfak2*, being phylogenetically positioned into group-1 insect AK family. *Ctenocephalides felis*-AK was largely soluble and not strongly associated with cellular structures. Besides adult fleas, AK was also detected in flea larvae and pupae. CfAK1 is a highly abundant protein, representing >3.5% of the soluble protein of adult fleas (Werr et al., 2009). The abundant levels of AK in unfed fleas might represent the involvement of this protein in cellular energy metabolism in association with the accumulation of energy resources in the larvae stage that are needed until blood feeding takes place.

### Ribosomal Proteins

In our study, ribosomal proteins, namely 60S and 40S, were predominant in several pairwise comparisons. Previously, when analyzing the sialotranscriptome of *X. cheopis* fleas, Andersen et al. (2007) found 20 proteins involved in protein synthesis (mostly ribosomal proteins), with two that were part of the proteasome machinery. The high abundance of ribosomal proteins in both unfed and fed fleas might be related to active protein synthesis in the adult unfed fleas a response to blood feeding.

### Proteasome Subunit α-Type

Proteasome subunit alpha type was predominant in fleas fed on uninfected cats for 24 hours when compared to unfed fleas and in fleas fed on uninfected cats for 9 days. The majority of regulated proteolysis in eukaryotes takes place *via* the ubiquitin/proteasome pathway, in which protein substrates are selected by the covalent attachment of multiple copies of the well-conserved polypeptide ubiquitin. After recognition and binding to a large complex (26S proteasome), the tagged protein is unfolded, loses the ubiquitin tag, and is then cleaved into small peptides. The proteasome’s core is a 20S particle comprising four stacked rings of seven subunits each; while the two outer rings are identical and consist of seven distinct, but related, α-type subunits, the two identical inner rings comprise seven different β-type subunits (Belote et al., 1998). Nguyen et al. (2019) demonstrated that the overexpression of the β5 subunit in adults of the *D. melanogaster* species does not result in transcriptional upregulation of the other subunits of the proteasome as they do in nematodes and human cell culture. Despite this lack of a regulatory role, boosting β5 expression increased the chymotrypsin-like activity associated with the proteasome, reduced both the size and number of ubiquitinated protein aggregates in aged flies, and increased longevity. Proteasome subunit α-type has been identified, in the sialome of the soft tick *Ornithodoros erraticus* (Oleaga et al., 2007) and *X. cheopis* (Andersen et al., 2007), by proteomic and transcriptomic approaches, respectively. Therefore, the abundance of Proteasome subunit alpha type in fleas fed on uninfected cats for 24 hours when compared to unfed and those fed for 9 days might be related to an intense cleavage of proteins during the first 24 hours of blood feeding.

### Juvenile Hormone Epoxide Hydrolase 1 (JHEH1)

JHEH1 had higher abundance levels in fleas fed on *B. henselae* uninfected cats for 24 hours when compared to both unfed fleas and those fed on uninfected cats for 9 days. While Juvenile hormone III (JH) is present during the flea larval stages in order to maintain the larval characters, its levels decrease in the last instar stage to allow metamorphosis and adult development. The production of JH is increased again in the adult cat flea, since it plays a major role in the regulation of flea reproduction by stimulating the differentiation of selected tissues in order to favor blood feeding and digestion, as well as vitellogenin synthesis (Meola et al., 2001; Keiser et al., 2002). Indeed, when exposed to increasing concentrations of JHIII, the adult cat flea ingested increasing amounts of blood and produced greater numbers of eggs over a 10 days period. The increase in cat flea fecundity associated with JH is due to a higher yolk protein synthesis by the fat body and uptake of vitellogenin by the maturing oocytes (Meola et al., 2001). However, while exposure of unfed adult fleas to high levels of JH caused cell membrane lysis and destruction of the fat body, midgut, salivary glands and ovaries, adult fed fleas presented autolysis and yolk resorption in the oocytes (Meola et al., 2001). Fine tuning degradation of JH is mediated by juvenile hormone esterase (JHE) (found in haemolymph and tissues), and by the tissue/membrane bound-JHEH1. While the former hydrolyses the ester of JH to JH-acid (JHA), the latter hydrolyses the epoxide of JH to JH-diol (JHD). The combined activities of both enzymes convert JH to JH- acid diol (JHAD), which is apparently non-active. The role that both enzymes play in JH degradation varies according to the stages of a certain insect species, as well as among different insect species (Keiser et al., 2002). JHEH is present in developing oocytes, fat body, and midgut epithelium of the adult cat flea, playing a role in the regulation of JHIII, so as to avoid deleterious levels and provide levels that halt feeding and vitellogenin production (Keiser et al., 2002). Keiser et al. (2002) suggested that JHEH might have a relatively minor role in JH regulation in the cat flea, when compared to JHE, since the latter was tenfold more active than the former in all flea life stages. However, JHE was not abundant in fleas in the present study. Therefore, the abundant levels of JHEH in fleas fed for 24 hours on uninfected control cat blood in the present study was potentially an attempt to avoid the deleterious effects of high levels of JH on the feeding, digestion and reproduction of adult cat fleas. Interestingly, vaccination of cats with JHEH1 resulted in the reduction of the number of viable cat flea females and decreased egg production (Contreras et al., 2018). Indeed, the effect of vaccination of cats with *C. felis* antigens (arginine kinase, Serpin 4, JHEH1, sodium/potassium-transporting ATPase subunit alpha, xylosyltransferase, and zinc transporter ZIP13 homolog) – of which three were abundant in fleas from the present study - on cat flea fecundity and egg hatchability could be explained by the potentially important role that these proteins play in metabolic and developmental processes in cat fleas (Contreras et al., 2018).

### Vitellogenin C

Vitellogenin C is a molecule synthesized in the fat body of insects, transported through the hemolymph until reaching female ovaries, where it is then taken up by oocytes. During vitellogenesis, it is responsible for enhancing yolk protein synthesis required for proper embryogenesis by maturing oocytes (Laukaitis and Macaluso, 2021). Indeed, Vitellogenin C levels were found to be approximately 9-fold higher in females compared with males (McIntosh et al., 2016). Juvenile hormone III plays a major role in regulating reproduction by processing of nutrients for vitellogenin synthesis (Meola et al., 2001). In the present study, Vitellogenin C was prominent in fleas fed on uninfected cats for both 24 hours and 9 days when compared to unfed fleas. Indeed, this protein had the highest fold change in fleas fed on uninfected cats for 24 hours when compared to unfed fleas. These findings might be related to demands for intense production of flea eggs during the first 24 hours of feeding, albeit the high levels at 9 days of feeding would also facilitate egg production. Interestingly, JHEH1 had higher abundance levels in Naïve Fleas fed for 24 hours, but not at 9 days. This may represent an attempt to regulate JHIII to levels that would then allow vitellogenin production to halt.

### Peritrophin-Like Protein (PL)

“Peritrophins” encompass proteins strongly bound to the peritrophic matrix, which is composed by proteins and sugar polymers (including chitin) that build up non-cellular linings in the gut of most insects. Peritrophins, which contain one or more putative chitin-binding (peritrophin-A) domains, may participate in the processes of digestion, nutrient and gas exchange, as well as protection against food particle-associated physical damage and infection (Tellam et al., 1999). Considering that adult *C*. *felis* do not produce a peritrophic matrix (Peters, 1992), these proteins are called “peritrophin-like” and may have roles other than those involved with the peritrophic matrix (Gaines et al., 2003). Previous analysis of expressed sequence tags from subtracted and unsubtracted adult *C. felis* cDNA libraries demonstrated that mRNAs for the peritrophin like protein 1 (PL1)-associated gene were exclusively expressed in the hindgut and Malpighian tubules tissues but not in the carcass (which included all body parts except the hindguts and Malpighian tubules) (Gaines et al., 2002). Later, immunostaining of the PL1 in the Malpighian tubules provided evidence that this protein is probably not associated with chitin *in vivo* (Gaines et al., 2002). Interestingly, even though the PL1 protein was immunostained in the chitinous cuticle from the trachea, hindgut, and rectum, it was indeed detected at higher levels in the Malpighian tubules, which in turn do not have a chitinous cuticle. The detection of PL1 in the trachea, hindgut and rectum supported the hypothesis that peritrophins and PL play a role in tissues with nutrient and gas exchange, as previously proposed (Tellam et al., 1999). Moreover, the authors speculated that PL1 might have a function other than chiting binding in the Malpighian tubules. These findings were corroborated by the chitin-binding assay, which showed that the PL1 protein derived from both the soluble and insoluble protein extracts failed to bind to chitin *in vitro*. Indeed, the presence of the PL1 in the insoluble protein extract evidenced that this protein may be associated with another kind of insoluble material besides chitin, such as insoluble glycoproteins (Gaines et al., 2003).

Similarly, when analyzing the transcriptomic profile of the digestive tract of *X. cheopis* following blood feeding and infection with *Y. pestis*, Bland et al. (2020) did not detect a thick peritrophic membrane by histochemistry or electron microscopy in rat flea guts in the first 24 hours of blood-feeding. Despite that, the authors identified six peritrophins in response to blood feeding, from which three (PL1, PL3, and peritrophic membrane protein 4) were more highly expressed in *Y. pestis*-infected fleas. In contrast, Peritrophin-like protein had higher abundance levels in unfed fleas when compared to fleas that fed on uninfected cats for 24 hours (PL1) and 9 days (PL1/PL2) and was not abundant in *B. henselae*-infected cat fleas in the present study. Interestingly, five *X. cheopis*-PL proteins identified by Bland et al. (2020) shared similarity with *C. felis*-PL and contained chitin binding Peritrophin-A domain. According to the authors, the increased expression of peritrophin A in *X. cheopis’* midgut, which lacks a membrane peritrophic, after a blood meal may represent a non-functional evolutionary leftover from Mecoptera, from which fleas diverged. Alternatively, the increased expression of peritrophin B in the trachea of *Y. pestis*-infected rat fleas might be a consequence of increased metabolism associated with tracheal pulsation due to *Y. pestis* infection, which in turn would initiate expression of peritrophins to repair the tracheal cuticle. In summary, flea peritrophin-like proteins may have several yet to be determined functions that are not associated with chitin binding.

### Allantoinase

Allantoinase is involved in the purine degradation pathway, in which it binds zinc ions and hydrolyses allantoin to form allantoic acid. As a final step, nitrogenous waste is produced and excreted (Gaines et al., 2002; Gaines et al., 2004). mRNAs for the allantoinase-coding gene was expressed exclusively in the hindgut tissues and Malpighian tubules in adult fleas but not in the carcass (which included all body parts except the hindguts and Malpighian tubules) (Gaines et al., 2002). Later, this enzyme was detected by immunohistochemistry in the same *C. felis* tissues (Gaines et al., 2004). Expression of the allantoinase-mRNA was detected by Northern blot in *C. felis* the first, third, and wandering larval stages, as well as in fed and unfed adults, but not in eggs or pupae (Gaines et al., 2004). In the present study, Allantoinase had higher abundance levels in fleas fed on uninfected cats for both 24 hours and 9 days when compared to unfed fleas. This finding might be associated with the flea blood feeding, which stimulated the purine degradation pathway due to the high ingestion of blood proteins.

### Phosphoenolpyruvate Carboxykinase (Pepck)

Upregulation of pepck is a common response of insects to environmental stress, such as heat in *D. melanogaster* and *Belgica antarctica* (Sørensen et al., 2005; Teets et al., 2013), cold in *Sarcophaga crassipalpis* (Teets et al., 2012; Teets et al., 2013), hypoxia in *D. melanogaster* (Liu et al., 2006), dehydration in *B. antarctica* (Teets et al., 2013), and oxidative stress in *D. melanogaster* (Girardot et al., 2004). Interestingly, pepck is strongly upregulated during diapause in *D. melanogaster* (Baker and Russell, 2009), *Sarcophaga crassipalpis* (Ragland et al., 2010), and *Rhagoletis pomonella* (Ragland et al., 2011), perhaps to upregulate glucose production in advance of adverse conditions. In the present study, pepck was predominant in fleas fed on *B. henselae*-infected cats compared to fleas fed on uninfected cats (both fed for 9 days), which might be related to the oxidative stress caused by *B. henselae* infection in the digestive tract of the cat flea.

### Succinic Semialdehyde Dehydrogenase (SSADH)

SSADH is a component of the γ-aminobutyric acid degradation pathway in mammals and is essential for development and function of the nervous system. The catabolic pathway for GABA includes two enzymes, namely GABA transaminase, which converts GABA to succinic semialdehyde (SSA), and SSADH, which oxidizes (NAD+-dependent) to succinate; whereby this latter metabolite enters the mitochondrial tricarboxylic acid cycle (Rothacker et al., 2008a; Rothacker et al., 2008b). *Ctenocephalides felis*-SSADH has been previously characterized (Rothacker et al., 2008a). While two introns are found in the *Lucilia cuprina* (blow fly) SSADH gene, no intron was present in the *C. felis* SSADH gene. Also, while one single copy of the SSADH gene is found in *Drosophila melanogaster* and mammals, multiple SSADH gene copies were observed in the genome of *L. cuprina, C. felis* and *Rhipicephalus microplus* (cattle tick) (Rothacker et al., 2008a; Rothacker et al., 2008b). SSADH is likely found in the mitochondrial matrix, considering the protein’s alkaline pH optimum and the presence of N-terminal polypeptides reminiscent of mitochondrial targeting sequences (Rothacker et al., 2008a). The GABA degradation pathway has been chosen as a molecular target group for new chemical compounds to combat ectoparasites (Rothacker et al., 2008b). In the present study, SSADH had the highest fold change in fleas fed on *B. henselae*-infected cats when compared to those fed on uninfected cats for 9 days. Based upon our results, we hypothesize that *B. henselae* infection and mutiplication in *C. felis* digestive tract triggers an additional production of energy in flea mitochondria.

### Glycinamide Ribotide Transformylase (GARTase)

In *D. melanogaster*, this protein is involved in step 2 of the subpathway that synthesizes 5-amino-1- (5-phospho-D-ribosyl) imidazole from N(2)-formyl-N(1)-(5-phospho-D-ribosyl)glycinamide. The purine *de novo* synthetic pathway consists of more than a dozen enzymatic activities leading to the synthesis of inosine monophlosphate (IMP) and other purine nucleotides. Indeed, these activities are performed by a large complex of several polypeptides (e.g. the multienzyme polypeptide GARTase). For instance, the *D. melanogaster* gene (Gart locus) is quite complex, encoding GARSase (glycinamide ribotide synthetase), AIRSase (aminoimidazole ribotide synthetase), and GARTase (Henikoff, 1986). In the present study, GARTase was predominant in fleas fed on uninfect cats for 24 hours and 9 days when compared to unfed fleas, which might be associated with the synthesis of purines that is stimulated by blood feeding.

### Secreted Salivary Acid Phosphatase

When analyzing the sialotranscriptome of fed *C. felis*, Ribeiro et al. (2012) found 81 ESTs corresponding to the phosphatase family, or nearly 17% of all ESTs of the S class (constituents of flea saliva). When comparing rat (*Xenopylla cheopis*) and cat flea phosphatases, the authors found an identity ranging from 21 to 84%, indicating divergence among these salivary proteins among different flea genera. Interestingly, the authors observed that 75% of the ESTs of the rat flea sialotranscriptome belonged to the S class, nearly 3 times the values found for cat flea sialotranscriptome (Ribeiro et al., 2012). Indeed, gene duplication events might have led to a large expansion of a family of acidic phosphatases that are probably inactive (Andersen et al., 2007). Hypotheses regarding such loss of function of flea acid phosphatases have been previously discussed (Andersen et al., 2007). Phosphorylated protein substrate might be the target of flea salivary phosphatases. According to Andersen et al. (2007), such loss of the enzymatic activity would maintain the substrate permanently blocked by an inactive interaction. Additionally, the loss of function of this group of enzymes would require larger amounts of the protein to interact with the target host protein, which might have been the reason for high levels of protein expression and gene duplication. Indeed, gene duplication may also favor avoiding immune detection by hosts. Lastly, the flea phosphatase family may also play a role in chelation of polyphosphates, which are released by platelets and are involved in preventing hemostasis (Andersen et al., 2007). In the present study, secreted salivary acid phosphase had higher abundance levels in fleas fed on *B. henselae*-infected cats for 9 days when compared to those fed on *B. henselae*-infected cats for 24 hours, which might be related to the maintenance of blood feeding by preventing hemostasis, as well as blockage of specific groups of phosphorylated protein substrates involved in the infection of fleas by *B. henselae*.

### FS-H/FSI Antigen Family Member 3

Previously, the sialotranscriptome of *X. cheopis* revealed transcripts coding for several peptides with similarity to a previously described antigen of the cat flea named FS-H precursor, as well as two other cat flea larger proteins named FS-I and antigen 1 precursors. The three disulfide-bond cysteine sequence motifs are characteristic of defensins, which are antimicrobial molecules important for protection against bacteria and fungi (Andersen et al., 2007). According to Andersen and colleagues, these peptides may act by controlling microbial growth or serve to induce analgesia at the site of the flea bite. Considering FS-H/FS-I antigen/7-Cys family of flea-specific peptides, Ribeiro et al. (2012) found seven members of this family when analyzing the sialotranscriptome of *C. felis*. Curiously, the alignment of the flea sequences showed an odd cysteine, which might be involved in redox reactions, in addition to a framework of six conserved cysteines, which in turn would be involved in three disulphide bonds, as previously observed (Andersen et al., 2007). Based on the unpaired cysteine, the authors speculated that these proteins might have an antioxidant function. In the present study, FS-H/FSI antigen family member 3 had higher abundance levels in unfed fleas when compared to fleas fed on *B. henselae*-infected cats for 9 days. This unexpected finding might be related to an antioxidant function in unfed fleas or alternatively protein inhibition of the antioxidant function by the bacteria.

### Elongation Factor 1-Alpha (EF-1α)

EF-1α, a homolog of EF-Tu in bacteria, is a nuclear protein-coding gene involved in the GTP-dependent binding of charged tRNAs to the acceptor site of the ribosome during translation (Danforth and Ji, 1998). EF-1α, a ubiquitously expressed protein that controls the efficiency and fidelity of mRNA translation in eukaryotic cells, is comprised of four subunits (α, β, γ, and δ) (Zhou et al., 2002). After binding aminoacyl-tRNAs, EF-1α transfers these molecules to 80 S ribosomes, while binding and hydrolyzing GTP. On the other hand, EF-1γ is associated with the β and δ subunits and stimulates the activity of EF- 1β in initiating the exchange of GDP to GTP on the α subunit (Zhou et al., 2002). EF-1α, which represents the most abundant component, may have several functions apart from protein synthesis (Schwientek et al., 2002). Changes in the level of EF-1α expression have indeed been implicated in aging, cytoskeletal reorganization, and ubiquitin-dependent proteolysis (Schwientek et al., 2002). Zhou et al. (2002) suggested that production of elongation factors, which were increased by juvenile hormone, might have contributed to the large protein synthesis required for egg production in *Locusta migratoria.* In this study, when Juvenile hormone epoxide hydrolase-1 was overrepresented in fleas fed on uninfected cats for 24 hours compared to unfed fleas, whereas EF-1α was not overrepresented.

In the present study, for the first time, flea differentially abundant proteins where characterized in response to both blood feeding and *B. henselae* infection. One limitation of the present study is the low number of biological and analytical replicates, which might have influenced the statistical power of the pairwise comparisons of the different experimental groups. Another limitation of this study is the fact that we worked with the whole flea proteome in response to *B. henselae* infection, instead of focusing only on the digestive tract protein abundance. Considering that *B. henselae* colonizes and multiplies in the digestive tract and is transmitted by flea feces, future studies based on proteomic and transcriptomic approaches aiming at investigating *C. felis* digestive tract’s proteins and transcripts in response to *B. henselae* infection may add important information to the results found in the present study. Despite these limitations, this study brings new and important information regarding the response of *C. felis* to blood feeding and *B. henselae* infection, often corroborating findings found in previous studies targeting fleas and other bacterial pathogens.

## Conclusions

Using proteomic approaches, the present work found that serine proteases, ribosomal proteins, proteasome subunit α-type, juvenile hormone epoxide hydrolase 1, vitellogenin C, allantoinase, phosphoenolpyruvate carboxykinase, succinic semialdehyde dehydrogenase, glycinamide ribotide transformylase, secreted salivary acid phosphatase had high abundance in response of *C. felis* blood feeding and/or infection by *B. henselae*. In contrast, high abundance of serpin-1, arginine kinase, ribosomal proteins, peritrophin-like protein, and FS-H/FSI antigen family member 3 was strongly associated with unfed cat fleas. Findings from this study provide insights into proteomic response of cat fleas to *B. henselae* infected and uninfected blood meal, as well as *C. felis* response to invading *B. henselae* over an infection time course, thus helping understand the complex interactions between cat fleas and *B. henselae* at protein levels. Further studies are needed to investigate the role of these identified differentially abundant cat flea proteins in response to infection with *B. henselae.* Finally, immunoproteomic analysis of the identified abundant cat flea proteins and evaluation of these proteins as candidate protective antigens for development of effective vaccines to control both *C. felis* infestations and *B. henselae* transmission require further studies.

## Data Availability Statement

The datasets presented in this study can be found in online repositories. The datasets can be found here: https://www.ncbi.nlm.nih.gov/nuccore/OK275541 and https://panoramaweb.org/NCSU%20-%20METRIC/METRIC%20Public%20Data/20210320%E2%80%94%E2%80%94CatFleaBartanella/project-begin.view.

## Ethics Statement

The animal study was reviewed and approved by Kansas State University KSU IACUC #4511-VMS; High Quality Research (HQR), Fort Collins, Colorado, USA, under an approved protocol (number #170.059).

## Author Contributions

MA, EBB, and RGM designed the study. EBB supervised and was responsible for funding acquisition. ML performed the experimental infection of cats with *Bartonella henselae*. BH and VS provided cat fleas for the experiment. TW, LC, and HB performed the proteomic techniques. PN performed the liquid-enrichment and isolation of *B. henselae* in blood agar plates from experimentally infected cats. JB performed the serological assays for *B. henselae*. MA performed the molecular techniques involved in the experiment. RGM performed ddPCR. TS and GJ performed the quantitative analysis of the abundant proteins. MA wrote the manuscript. All authors revised and contributed to the article and approved the submitted version.

## Funding

This research was supported by the state of North Carolina and through donations to the *Bartonella*/Vector Borne Diseases Research Fund at the North Carolina State University College of Veterinary Medicine Foundation and to the Center for Companion Animal Studies at Colorado State University. The authors would like to acknowledge Fundação de Amparo à Pesquisa do Estado de São Paulo (FAPESP Process 2019/09464-6) for the sabbatical fellowship conceived to MRA at North Carolina State University. MRA is a fellowship of CNPq (Conselho Nacional de Desenvolvimento Científico e Tecnológico - Productivity Grant Process #302420/2017-7). This work was performed in part by the Molecular Education, Technology and Research Innovation Center (METRIC) at NC State University, which is supported by the State of North Carolina.

## Conflict of Interest

RGM is a co-founder and the Chief Technical Officer for Galaxy Diagnostics Inc. In conjunction with Dr. S. Sontakke and North Carolina State University, EBB holds US Patent No. 7,115,385 Media and Methods for Cultivation of Microorganisms, which was issued on October 3rd, 2006. He is a co-founder, shareholder and Chief Scientific Officer for Galaxy Diagnostics, a company that provides advanced diagnostic testing for the detection of *Bartonella* spp. infections.

The remaining authors declare that the research was conducted in the absence of any commercial or financial relationships that could be construed as a potential conflict of interest.

## Publisher’s Note

All claims expressed in this article are solely those of the authors and do not necessarily represent those of their affiliated organizations, or those of the publisher, the editors and the reviewers. Any product that may be evaluated in this article, or claim that may be made by its manufacturer, is not guaranteed or endorsed by the publisher.
