# Supplementary material for: Using Proteomic Approaches to Unravel the Response of Ctenocephalides felis felis to Blood Feeding and Infection With Bartonella henselae
Source: Front Cell Infect Microbiol. 2022 Jan 28;12:828082. doi: 10.3389/fcimb.2022.828082 (PMC8831700; doi:10.3389/fcimb.2022.828082)
Supplement: Supplementary file 1 [file Table_1.docx]

**Supplementary Material (SM)**

**Table SM1.** Summarized results of serological (IFAT), molecular (qPCR based on ITS) and microbiological (enrichment liquid medium BAPGM; blood agar culturing) assays applied on one sterile saline-inoculated cat (#3363) blood samples and adult fleas and flea dirt collected 24 hours and 9 days feeding.

| **IFAT** | **Day 0** | **Day 7** | **Day 14** | **Day 21** | **Day 28** | **Day 29** | **Day 37** |
| --- | --- | --- | --- | --- | --- | --- | --- |
|  | 64 | 16 | 16 | 32 | 64 |  | 32 |
| **qPCR (ITS) blood sample** | - | - | - | - | - |  | - |
| **Blood agar plate** |  |  |  |  | No colonies |  | No colonies |
| **BAPGM C7-Blood agar plate** |  |  |  |  | qPCR –  No colonies |  | qPCR –  No colonies |
| **BAPGM C14-Blood agar plate** |  |  |  |  | qPCR –  No colonies |  | qPCR –  No colonies |
| **BAPGM C21-Blood agar plate** |  |  |  |  | qPCR –  No colonies |  | qPCR –  No colonies |
| **qPCR**  **fleas** |  |  |  |  |  | - | - |
| **qPCR flea dirt** |  |  |  |  |  | - | - |

Day 0: Inoculation of the cat #3363 with sterile saline; Day 7: one week after inoculation; Day 14: two weeks after inoculation; Day 21: three weeks after inoculation; Day 28: four weeks after inoculation with saline when flea infestation was performed; Day 29: one of the chambers were removed from the cat after 24 hours of flea feeding; Day 37: the remaining chambers were removed from the cat after 9 days of flea feeding; +: positive; -:negative; Cq: Cycle of quantification; CFU/mL: colony forming unit per microliter.

**Table SM2.** Summarized results of serological (IFAT), molecular (qPCR based on ITS) and microbiological (enrichment liquid medium BAPGM; blood agar culturing) assays applied on one sterile saline-inoculated cat (#3508) blood samples and adult fleas and flea dirt collected 24 hours and 9 days feeding.

| **IFAT** | **Day 0** | **Day 7** | **Day 14** | **Day 21** | **Day 28** | **Day 29** | **12/03/20** |
| --- | --- | --- | --- | --- | --- | --- | --- |
|  | 128 | 32 | 64 | 64 | 128 |  | 128 |
| **qPCR blood sample** | - | - | - | - | - |  | - |
| **Blood agar plate** |  |  |  |  | No colonies |  | No colonies |
| **BAPGM C7-Blood agar plate** |  |  |  |  | qPCR –  No colonies |  | qPCR –  No colonies |
| **BAPGM C14-Blood agar plate** |  |  |  |  | qPCR –  No colonies |  | qPCR –  No colonies |
| **BAPGM C21-Blood agar plate** |  |  |  |  | qPCR –  No colonies |  | qPCR –  No colonies |
| **qPCR fleas** |  |  |  |  |  | - | - |
| **qPCR flea dirt** |  |  |  |  |  | - | - |

Day 0: Inoculation of the cat #3508 with sterile saline; Day 7: one week after inoculation; Day 14: two weeks after inoculation; Day 21: three weeks after inoculation; Day 28: four weeks after inoculation with saline when flea infestation was performed; Day 29: one of the chambers were removed from the cat after 24 hours of flea feeding; Day 37: the remaining chambers were removed from the cat after 9 days of flea feeding; +: positive; -:negative; Cq: Cycle of quantification; CFU/mL: colony forming unit per microliter.

**Table SM3.** Summarized results of serological (IFAT), molecular (qPCR based on ITS and cPCR based on ITS and *gltA*) and microbiological (blood agar culturing) assays applied on one *Bartonella henselae*-experimentally infected cat (#3320) blood sample and adult fleas and flea dirt collected 24 hours and 9 days feeding.

| **IFAT** | **Day 0** | **Day 7** | **Day 14** | **Day 21** | **Day 28** | **Day 29** | **Day 37** |
| --- | --- | --- | --- | --- | --- | --- | --- |
|  | 1:128 | 1:128 | 1:256 | 1:512 | 1:1024 |  | 1:1024 |
| **qPCR (ITS) blood sample** | - | +  (Cq = 34.62) | +  (Cq= 23.9) | +  (Cq= 22.03) | +  (Cq= 31.56) |  | +  (Cq=32.44) |
| **cPCR blood sample (*gltA* 850s/1120as)** | - | + | + | + | + | + | + |
| **cPCR blood sample (ITS 325s/ 1100as)** | - | + | + | + | + | + | + |
| **Blood agar plate** |  |  |  |  | 2.4 x 10^4^ CFU/mL |  | 2 x 10^3^ CFU/mL |
| **qPCR (ITS) fleas** |  |  |  |  |  | +  (Cq = 38.13) | +  (Cq = 41.48) |
| **cPCR fleas (*gltA* 850s/1120as)** |  |  |  |  |  | + | - |
| **cPCR fleas (ITS 325s/ 1100as)** |  |  |  |  |  | + | - |
| **qPCR (ITS) flea dirt** |  |  |  |  |  | +  (Cq = 39.17) | +  (Cq = 34.83) |
| **cPCR flea dirt (*gltA* 850s/ 1120as)** |  |  |  |  |  | - | + |
| **cPCR flea dirt (ITS 325s/1100as)** |  |  |  |  |  | - | + |

Day 0: Experimental infection of cat #3320 with *B. henselae*; Day 7: one week after experimental infection; Day 14: two weeks after experimental infection; Day 21: three weeks after experimental infection; Day 28: four weeks after experimental infection when flea infestation was performed; Day 29: one of the chambers were removed from the cat after 24 hours of flea feeding; Day 37: the remaining chambers were removed from the cat after 9 days of flea feeding; +: positive; -:negative; Cq: Cycle of quantification; CFU/mL: colony forming unit per microliter. Conventional PCR assays were run for previous qPCR-positive samples.

**Table SM4.** Summarized results of serological (IFAT), molecular (qPCR based on ITS and cPCR based on ITS and *gltA*) and microbiological (blood agar culturing) assays applied on one *Bartonella henselae*-experimentally infected cat (#3711) blood sample and adult fleas and flea dirt collected 24 hours and 9 days feeding.

| **IFAT** | **Day 0** | **Day 7** | **Day 14** | **Day 21** | **Day 28** | **Day 29** | **Day 37** |
| --- | --- | --- | --- | --- | --- | --- | --- |
|  | 1:32 | 1:16 | <1:16 | 1:512 | 1:1024 |  | 1:512 |
| **qPCR (ITS) blood sample** | - | +  (Cq = 37.45) | +  (Cq= 22.79) | +  (Cq= 23.19) | +  (Cq= 26.17) |  | +  (Cq=28.09) |
| **cPCR blood sample (*gltA* 850s/1120as)** | - | + | + | + | + | + | + |
| **cPCR blood sample (ITS 325s/1100as)** | - | + | + | + | + | + | + |
| **Blood agar plate** |  |  |  |  | 1.4 x 10^5^ CFU/mL |  | 5.8 x 10^4^ CFU/mL |
| **qPCR fleas** |  |  |  |  |  | +  (Cq = 31.61) | +  (Cq = 32.48) |
| **cPCR fleas (*gltA* 850s/1120as)** |  |  |  |  |  | + | + |
| **cPCR fleas (ITS 325s/1100as)** |  |  |  |  |  | + | + |
| **qPCR flea dirt** |  |  |  |  |  | +  (Cq = 31.03) | +  (Cq = 29.06) |
| **cPCR flea dirt (*gltA* 850s/1120as)** |  |  |  |  |  | + | + |
| **cPCR flea dirt (ITS 325s/1100as)** |  |  |  |  |  | + | + |

Day 0: Experimental infection of cat #3711 with *B. henselae*; Day 7: one week after experimental infection; Day 14: two weeks after experimental infection; Day 21: three weeks after experimental infection; Day 28: four weeks after experimental infection when flea infestation was performed; Day 29: one of the chambers were removed from the cat after 24 hours of flea feeding; Day 37: the remaining chambers were removed from the cat after 9 days of flea feeding; +: positive; -:negative; Cq: Cycle of quantification; CFU/mL: colony forming unit per microliter. Conventional PCR assays were run for previous qPCR-positive samples.

**Table SM5.** Protein concentration (mg/mL) of each experimental group comprising two biological replicates with 320 fleas each.

| **Experimental Groups** | **Protein Concentration (mg/mL)** |
| --- | --- |
| **Group 1 – Unfed Fleas** |  |
| Sample 1 | 1,044 |
| Sample 2 (Biological Replicate for Group 1) | 1,174 |
| **Group 2 – Fleas fed on uninfected cats for 24 hours** |  |
| Sample 3 – Fleas fed on cat #3363 | 1,730 |
| Sample 4 - Fleas fed on cat #3508 (Biological Replicate for Group 2) | 1,697 |
| **Group 3 – Fleas fed on uninfected cats for 9 days** |  |
| Sample 5 – Fleas fed on cat #3363 | 3,204 |
| Sample 6 - Fleas fed on cat #3508 (Biological Replicate for Group 3) | 1,721 |
| **Group 4 – Fleas fed on *B. henselae*-infected cats for 24 hours** |  |
| Sample 7 – Fleas fed on cat #3320 | 3,288 |
| Sample 8 - Fleas fed on cat #3711 (Biological Replicate for Group 4) | 1,887 |
| **Group 5 – Fleas fed on *B. henselae*-infected cats for 9 days** |  |
| Sample 9 – Fleas fed on cat #3320 | 2,850 |
| Sample 10 - Fleas fed on cat #3711 (Biological Replicate for Group 5) | 2,922 |
